# 4D Biofabrication of Magnetically Augmented Callus Assembloid Implants Enables Rapid Endochondral Ossification via Activation of Mechanosensitive Pathways

**DOI:** 10.1002/advs.202413680

**Published:** 2025-02-25

**Authors:** Konstantinos Ioannidis, Andreas Dimopoulos, Isaak Decoene, Maya Guilliams, Hanna Svitina, Liudmyla Storozhuk, Rodrigo de Oliveira‐Silva, Sergey Basov, Nguyen Thi Kim Thanh, Stefanos Mourdikoudis, Margriet J. Van Bael, Bart Smeets, Dimitrios Sakellariou, Ioannis Papantoniou

**Affiliations:** ^1^ Prometheus Translational Division of Skeletal Tissue Engineering KU Leuven, O&N1, Herestraat 49, PB 813 Leuven 3000 Belgium; ^2^ Skeletal Biology and Engineering Research Centre, Department of Development & Regeneration KU Leuven O&N1, Herestraat 49, PB 813 Leuven 3000 Belgium; ^3^ Healthcare Biomagnetics and Nanomaterials Laboratories, Department of Medical Physics and Biomedical Engineering University College London 21 Albemarle Street London W1S 4BS UK; ^4^ London Centre for Nanotechnology University College London 17‐19 Gordon Street London WC1H 0AH UK; ^5^ MeBioS division, Biosystems Department KU Leuven Kasteelpark, Arenberg 30 Leuven 3001 Belgium; ^6^ Membrane Separations, Adsorption, Catalysis, and Spectroscopy for Sustainable Solutions (cMACS), Department of Microbial and Molecular Systems KU Leuven Celestijnenlaan 200F, PB 2454 Leuven 3001 Belgium; ^7^ Quantum Solid State Physics, Department of Physics and Astronomy KU Leuven Celestijnenlaan 200D Leuven 3001 Belgium; ^8^ Biophysics Group, Department of Physics and Astronomy University College London Gower Street London WC1E 6BT UK; ^9^ CINBIO, Department of Physical Chemistry, Campus Universitario, Lagoas Marcosende Universidade de Vigo Vigo 36310 Spain

**Keywords:** 4D Biofabrication, bone, cartilaginous microtissues, magnetic actuation, magnetic nanoparticles, magnetic stimulation, regeneration

## Abstract

The use of magnetic‐driven strategies for non‐contact manipulation of engineered living modules opens up new possibilities for tissue engineering. The integration of magnetic nanoparticles (MNPs) with cartilaginous microtissues enables model‐driven 4D bottom‐up biofabrication of remotely actuated assembloids, providing unique properties to mechanoresponsive tissues, particularly skeletal constructs. However, for clinical use, the long‐term effects of magnetic stimulation on phenotype and in vivo functionality need further exploration. Magnetic‐driven biofabrication includes both rapid processes, such as guided microtissue assembly, and slower biological processes, like extracellular matrix secretion. This work explores the interplay between magnetic fields and MNP‐loaded cartilaginous microtissues through mathematical modeling and experimental approaches, investigating long‐term stimulation effects on ECM maturation and chondrogenic hypertrophy. Transcriptomic analysis reveal that magnetic stimulation activated mechanosensitive pathways and catabolic processes, driving accelerated cartilage‐to‐bone transitions via endochondral ossification, outcomes not observed in non‐stimulated controls. This study paves the way for pre‐programmed, remotely actuated skeletal assembloids with superior bone‐forming capacity for regenerating challenging bone fractures.

## Graphical abstract

Magnetically augmented biohybrid cartilaginous microtissues enable remote microtissue motion control and assembly as well as targeted differentiation. This study demonstrates accelerated endochondral ossification under magnetic stimulation, highlighting cartilaginous assembloid constructs’ ability to retain long‐term magnetic properties. Magnetic microtissues can be used in the future as injectables for minimally invasive approaches enabling in situ biofabrication and accelerated skeletal repair.

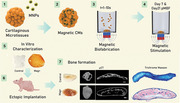

## 1 Introduction

Recent years have seen a rapid growth in the development of magnetically responsive living systems for applications in tissue engineering and regenerative medicine.^[^ ^1^, ^2^ ^]^ Recent studies have explored remote control of cell laden hydrogels by embedding superparamagnetic iron oxide nanoparticles (SPIONs), hereafter referred to as magnetic nanoparticles (MNPs), are enabling remote motion control,^[^ ^3^, ^4^ ^]^ actuation capabilities^[^ ^5^ ^]^ and multiple cellular functionalities^[^ ^6^ ^]^ related to tissue engineering systems.^[^ ^7^ ^]^ This has been achieved either through direct contact of MNPs with cells^[^ ^8^ ^]^ or via embedding of MNP in polymer fibers^[^ ^9^ ^]^ and hydrogel carriers.^[^ ^10^ ^]^ Moreover, great interest has been shown on the use of magnetic fields for biofabrication strategies utilizing spheroid building blocks with MNPs.^[^ ^11^, ^12^, ^13^ ^]^ Magnetically guided biofabrication methods and setups have used MNPs to establish and develop living material bioassemblies with inherent magnetic properties able to undergo external actuation and guidance.^[^ ^8^, ^14^, ^15^, ^16^ ^]^ Moreover, magnetic forces have been shown to drive morphogenetic events and patterning in organoids recapitulating early developmental processes through targeted mechanical stimulation via the incorporation of MNPs.^[^ ^17^ ^]^ This demonstrates that magnetic forces can enable rapid motion and shape morphing but also dictate slower biological processes over time. Despite extensive exploration on the influence of magnetic fields on the locomotory and actuation aspects of tissue engineered constructs, there is currently ample room to explore long‐term evaluation of magnetic stimulation on tissue and differentiation properties as well as their in vivo functionality.

“Bottom‐up” tissue engineering approaches permit the design of more precise biological hierarchy while allowing to build‐in predefined phenotypic features using organoid and microtissue building blocks.^[^ ^18^ ^]^ Organoids recapitulate complex in vivo processes of the corresponding native tissues providing valuable building blocks for tissue biofabrication, with preprogrammed biological properties and predictable functionality.^[^ ^19^, ^20^ ^]^ Several features such as the shape and composition of individual blocks can be easily controlled, thereby facilitating the development of versatile tissue structures.^[^ ^21^ ^]^ Furthermore, this approach enables the biofabrication of engineered tissues with high‐cell density^[^ ^22^, ^23^, ^24^, ^25^, ^26^ ^]^ which has been progressively seen as a critical factor for functionality post implantation.

Bone tissue engineering is evolving toward the biomimicry of the regenerative fracture healing process, which mimics embryonic development and long bone formation,^[^ ^27^, ^28^ ^]^ and which is mediated by the formation of a cartilage intermediate tissue that experiences endochondral ossification in order to heal the bone fracture.^[^ ^29^ ^]^ Recent studies have demonstrated that following bottom‐up tissue engineering strategies, functional cartilaginous assembloids could be engineered which were able to autonomously undergo bone organogenesis and result in long bone fracture healing.^[^ ^27^, ^28^, ^30^, ^31^ ^]^ Although self‐assembled implants have demonstrated great potential in skeletal tissue engineering and bone regeneration,^[^ ^32^, ^33^ ^]^ these cellular assemblies are still based on self‐assembly and still face critical limitations regarding their biofabrication into more controlled implants. Despite the development of novel biofabrication processes designed for spheroids and microtissues,^[^ ^30^, ^32^ ^]^ there is still a gap in obtaining adequate control over slower biological occurring processes such as extra cellular matrix (ECM) secretion and phenotype transitions during differentiation. This issue becomes critical when considering further scale‐up and standardization, thus hindering the predictability of final tissue properties and limiting their clinical applicability. The use of cartilaginous microtissue as building blocks for bone tissue engineering is a rapidly growing domain^[^ ^30^ ^]^ due to its promising results and biomimicry of the cartilage to bone transition observed during fracture healing.^[^ ^34^ ^]^ Cartilaginous microtissues derived from various adult progenitor cell types such as bone marrow MSCs,^[^ ^35^ ^]^ adipose derived MSCs^[^ ^36^ ^]^ and hPDCs^[^ ^32^, ^37^ ^]^ have been to date validated for their capacity to undergo endochondral ossification upon implantation in small animal models. Some of these studies demonstrated even the potential of this strategy for regeneration of critical size tibial^[^ ^38^ ^]^ and cranial defects.^[^ ^39^ ^]^ Moreover, cartilaginous microtissues can be merged with biomaterials such as melt electrowritten meshes^[^ ^40^ ^]^ and hydrogels^[^ ^41^ ^]^ in order to produce microtissue‐hydrogel hybrids. However, bioprinting of microtissue suspensions can be challenging due to high shear stress exposure during flow through the nozzle.^[^ ^42^ ^]^ In addition, this method does not allow the development of engineered implants with high density of microtissues.^[^ ^43^ ^]^ Therefore, novel low shear biofabrication strategies enabling the development of high‐density cartilaginous implants need to be further explored. Recognizing this gap, we aim to provide an extensive study on the in vivo performances of magnetized skeletal implants.

In this work, we present the development of magnetically responsive next‐generation skeletal assembloid implants following 4D biofabrication in the context of a developmental engineering strategy.^[^ ^21^, ^32^, ^44^ ^]^ We initially formed microtissues using human progenitor skeletal cells from the periosteum and allowed them to go through the initial proliferative phase which corresponds to the initial phase of fracture healing.^[^ ^45^ ^]^ Subsequently we incorporated MNPs on these microtissues and by exposure to a magnetic field we enabled the coalescence of a microtissues populations and the formation of a “callus assembloid” construct. This assembloid was remotely stimulated in a static magnetic field during further chondrogenic differentiation for a long time period of up to 21 days. Magnetic callus assembloids (MCAs) were able to maintain their magnetic properties throughout the differentiation period and exhibited responsiveness to the magnetic field through shape forming but also via microstructural ECM adaptation and distinct phenotype acquisition. Collectively these properties resulted in strongly improved in vivo bone properties of these novel MCA implants. Our in vivo data revealed that endochondral ossification was strikingly accelerated in response to non‐stimulated control assembloids due to prior magnetic stimulation. These findings pave the way to the design of magnetically augmented cartilaginous implants that address bottlenecks for challenging bone defects in the future.

## 2 Results

### 2.1 Generation of Magnetic Callus Assembloids

**Figure 1:**
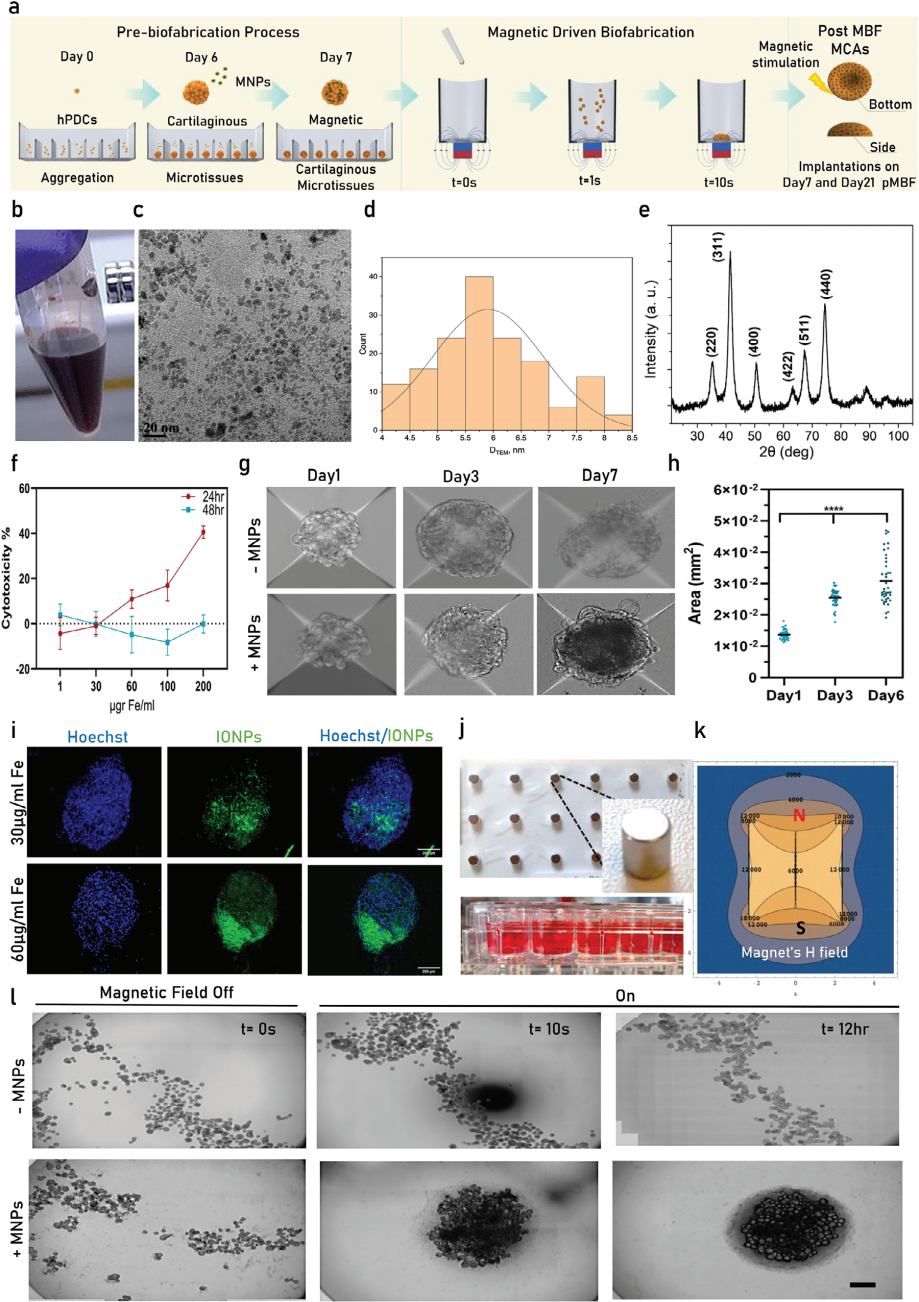
Generation of magnetic augmented callus microtissues for magnetic‐guided bioassembly. a) Schematic overview of the magnetic biofabrication process: cellular aggregation (Day 0), condensation (Day 1), differentiation (Day 6), and subsequent MNP incorporation followed by 24‐h incubation (Day 7). This was followed by the formation of callus assembloids upon exposure to a magnetic field. Samples pMBF were cultured for an additional 7 or 21 days under magnetic stimulation and subsequently analyzed or used for in vivo implantations. b) Photo image showing the MNPs dispersion used in this study. c) TEM image of MNPs d) Size distribution plot of MNPs showing an average of 6 nm particle size e) XRD diffraction spectrum of MNPs indicating the characteristic peaks of iron oxide. f) Cytotoxicity (MTT) percentage plot for a range of iron loading performed after 1 day of incubation with MNPs (*N* = 3). g) Microscope images showing the generation of magnetic and non‐magnetic microtissues on Day 7 (*N* = 3). h) Plot measuring the increasing area size of cartilaginous microtissues during in vitro maturation. i) Fluorescent microscope images of different fluorescein conjugated MNPs concentrations reveal successful incorporation of MNPs within the microtissues. Scale bar: 200 µm. j) Photo showing the magnetic grid plate used for the magnetic assembly k) Magnetic field plot of a neodymium magnet i) Microscope images showing the responsiveness of magnetized and non‐magnetized microtissues upon exposure to external static magnetic field. Scalebar: 1 mm.

In order to generate magnetically‐augmented cartilaginous microtissues we first developed size‐controlled microaggregates and initiated chondrogenic differentiation for 7 days as previously demonstrated.^[^ ^32^ ^]^ In this period cells self‐assemble, condense and initiate the secretion of extracellular matrix forming cartilaginous microtissues of early maturity. Following that initial step, microtissues were incubated with MNPs and were subsequently used for magnetic‐guided biofabrication of MCAs as well as long term magnetic stimulation as shown in the overview schematic **Figure** **1**a. The MNPs used in this study have been extensively characterized previously after their synthesis and have been demonstrated as biocompatible agents for biomedical applications.^[^ ^46^ ^]^ Essentially our MNPs are SPIONs (Figure 1b) coated with citric acid and possess an average particle size diameter of 6 nm (Figure 1d), measured after obtaining transmission electron microscopy (TEM) images from MNPs dispersions, see Figure 1c. XRD diffractogram of citric acid coated iron oxide nanoparticles showed diffraction peaks at 2θ values of ≈27.1°, 43.1°, 45.1°, 62.0°, 65.0°, and 72.6°, which correspond to the (220), (311), (400), (422), (511), and (440) lattice planes of the inverse spinel structure according to the ICDD (previously JCPDS) pattern number #03‐065‐3107 indicating the presence of magnetite (Fe_3_O_4_) as illustrated in Figure 1e.

Prior to incorporating MNPs to microtissues, their cytotoxicity was assessed using an MTT assay on the cellular level (2D) after 24 and 48 h of incubation, with the percentage of cytotoxicity normalized against measurements from untreated cells after the corresponding time of incubation with the MNPs. Cells were incubated with varying iron concentrations (1, 30, 60, 100, 200 µg Fe per mL). Cytotoxicity began to increase when the Fe_3_O_4_ concentration exceeded 60 µg of Fe per mL, with significant effects observed at 100 and 200 µg Fe per mL. In contrast, lower concentrations (1, 30, and 60 µg Fe per mL) did not cause any remarkable cytotoxic effect or metabolic stress (Figure 1f). After 48 h of incubation, the metabolically stressed samples with increased Fe uptake exhibited no signs of cytotoxicity. Instead, they demonstrated enhanced metabolic activity compared to the untreated control samples at the corresponding timepoint. Following integration with MNPs, microtissues were monitored throughout the chondrogenic differentiation phases using optical microscopy (Figure 1g). Their growth in size and area was quantified during culture by analyzing their area distribution, as illustrated in the plot in Figure 1h.

Microscope images on Figure S3, Supporting Information, particularly Figure S3a, Supporting Information showcase the different MNPs incorporating potential depending on Fe loading and lack of structural ECM in the matrix of microtissues. This prolonged treatment did not compromise cell viability, as demonstrated in Figure S3b, Supporting Information using Live/Dead staining, when Day 7 microtissues were utilized for the MBF process. In addition, in Figure S3, Supporting Information, we performed a detailed study to assess the impact of iron loading on 3D microtissues undergoing cartilaginous differentiation using LDH assay. While the MTT assay was initially performed on 2D cultures to assess the baseline metabolic activity and potential cytotoxicity of the MNPs, this may not fully reflect the 3D microtissue environment. To address this limitation, we complemented the MTT assay with an LDH release assay on 3D microtissues, which provides a more direct measure of membrane integrity and cell viability. Interestingly, our LDH assay results demonstrated that untreated samples exhibited higher LDH release compared to those treated with MNPs (Figure S3c, Supporting Information), particularly at higher concentrations (60 µg Fe) over time without affecting cell viability (Figure S3b, Supporting Information). This indicates that MNP‐treated microtissues experienced reduced cell death and membrane disruption. As a positive control, we incubated Day 7 microtissues with 1% Triton X, which served as the maximum LDH release measurement in our system (Figure S5d, Supporting Information). Furthermore, we performed reactive oxygen species (ROS) assay on 2D‐cultured hPDCs after 24 h of incubation, which revealed decreased ROS levels with increasing Fe concentrations (Figure S3e, Supporting Information). These findings underscore the biocompatibility and antioxidant properties of MNPs in the 3D system.

Furthermore, the incorporation of MNPs within the microtissues was confirmed through fluorescent imaging at two different iron loading concentrations (30 and 60 µg of Fe mL^−1^). Fluorescein (green)‐labeled MNPs were used to track and localize their presence on the microtissue surface, as depicted in Figure 1i. Moreover, microtissues magnetized at these iron loadings exhibited a rapid response when exposed to a strong static magnetic field (Figure 1k) generated by a magnetic grid (Figure 1j) made of cylindrical NdFeB permanent magnets (5mm diameter and 5 mm height, Brmax: 14800 Gauss). The magnetic field (max 0.4T measured at the bottom of the well) provided by the magnetic grid, guided the bioassembly of magnetic cartilaginous microtissues (MCAs, Figure 1l) in a single biofabrication step lasting less than 10 s, resulting in an implant of similar dimensions (5 mm) to the magnet.

### 2.2 Magnetic Set‐Up Characterization and Development of a Computational Model for Studying the Magnetic‐Guided Assembly

As demonstrated, the permanent neodymium magnet provided sufficient magnetic strength to transport microtissues, facilitating their assembly into an assembloid construct through the interaction of non‐uniform MNP gradients and magnetic field gradients. Therefore, we aimed to fully characterize our magnetically driven assembly setup and develop a computational model that incorporates the system's biophysical parameters to build a tool capable of predicting the assembly kinetics of MNP‐loaded microtissues with varying MNP loadings.

**Figure 2:**
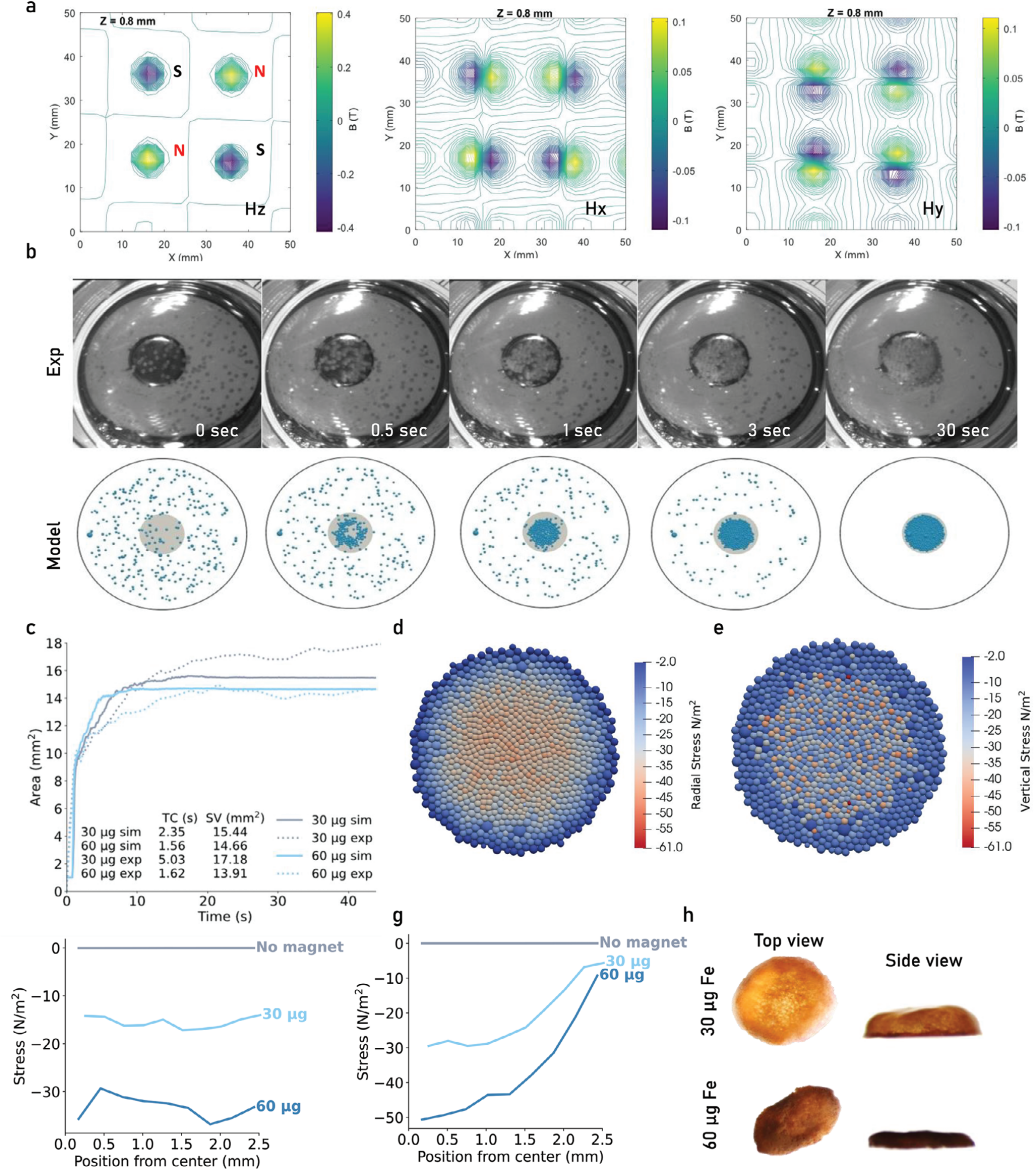
Characterization of the Magnetic Set‐Up and MBF model. a) 3D magnetic field mapping (Hz, Hx and Hy) of the grid using a Hall probe) for 4 neighboring magnets and the corresponding magnetic field directions. b) Stereoscope video recording images of the experimental bioassembly (first row) and images from the simulation model developed to predict the bioassembly (second row) using 30 µg of Fe per ml. c) Assembloid area coverage and time coefficient plot of the computational bioassembly process using our system parameters and data extracted from the computational model. d) Radial and e) Vertical stress distribution across MCAs. f) Quantification of radial stress for no magnet, 30 and 60 µg of Fe. g) Quantification of vertical stress for no magnet, 30 and 60 µg of Fe. h) Stereoscope images showing top, bottom and side view of 30 and 60 µg loaded and stimulated samples after 1 week in culture.

Initially, to analyze and map the magnetic field we employed 3D scanning and mapping techniques. This analysis was carried out for a magnetic field generated by a grid of four magnets positioned at the center of the magnetic plate across the x, y, and z axes, using a Hall probe, as depicted in **Figure** **2**a. These measurements were crucial for identifying the conditions that guided microtissue assembly while suggesting insights into spatial variation and gradient strength (Figure S4, Supporting Information). Moreover, these values were essential for the development of a computational model able to simulate the magnetic‐guided bioassembly process. To achieve this, we conducted experiments to collect data on the physical properties of microtissues, including size, weight, and stiffness as provided in **Table** **1**. Furthermore, we integrated the magnetic field equation into our computational model, which characterized a cylindrical magnet with specifications matching those of our setup, alongside varying MNPs loading conditions. To validate the accuracy of the model, we conducted experiments where we recorded videos of the bioassembly process using a stereoscope (Video S1, Supporting Information), allowing us to extract and compare experimental observations with model predictions recordings (Figure 2b, Video S2, Supporting Information). Our analysis focused on interpreting the behavior of microtissues in the magnetic field and the dynamics of biofabrication area over time for different MNPs loadings. Our findings demonstrate that the computational model accurately predicted the timing and morphology of the bioassembly process matching closely experimental observations.

**Table 1:** Model parameters.

Parameter	Value	Unit
Timestep [Δt]	5 × 10⁻⁵	s
Magnetic permeability of vacuum [μ₀]	1.256 × 10⁻⁶	N A^−^^2^
Relative magnetic permeability of water [µ_r_]	1	–
Gravitational constant [g]	9.807	m s^−^^2^
Viscosity of the medium [η_m_]	1.200 × 10⁻^3^	Pa s
Density of the medium [ρ_m_]	1000	kg m^−^^3^
Density of the spheroid [ρ_s_]	1014	kg m^−1^ ^3^
Stiffness of the well [E_s_]	1000	Pa
Adhesion energy density between spheroid and well [σ̂]	10⁻^2^⁰	N m^−1^
Tangential contact damping for spheroid‐well contact [c_t_]	0.6	Pa s m^−1^
Normal contact damping for spheroid‐well contact [c_n_]	0.6	Pa s m^−1^
Elastic modulus of the spheroid [E_s_]	120	Pa
Simulation time [t_end_]	60	s
Total mass of MNP added 30 µg [*m*_NPtot_]	10	µg
Total mass of MNP added 60 µg [*m*_NPtot_]	20	µg
Output interval [I_out_]	1	s
Degree of implicitness [λ_i_]	0	–
MNP absorption [α]	0.75	–

As anticipated, the differential loading of MNPs had a significant impact on bioassembly kinetics, as evidenced by experimental results comparing microtissues loaded with 30 µg of Fe (experimental: 5.03s, model: 2.35s) and 60 µg of Fe (experimental: 1.62s, model: 1.56s) (Figure 2c). Building on the validated model, we expanded our analysis to incorporate tissue assembly dynamics, allowing us to visualize and quantify both radial stresses (Figure 2d) and vertical stresses (Figure 2e) experienced by microtissues within the magnetic cell aggregates (MCAs) during the magnetic‐driven bioassembly process. In Figure 2f, the results of the radial stress quantification analysis reveal a negative stress, indicating a compressive magnetic force (FmagMax = −30 N m^−^^2^ for 30 µg Fe and −52 N m^−^^2^ for 60 µg Fe), which diminishes as distance increases from the center of the magnet. Figure 2 g presents the results of the vertical stress quantification analysis, which similarly shows a compressive stress (−15 N m^−^^2^ for 30 µg Fe and −32 N m^−^^2^ for 60 µg Fe). Unlike radial stress, vertical stress retains a consistent magnitude across distances from the magnet center, implying a consistent downward force acting on the microtissues. Following the magnetic‐guided biofabrication process, the MCAs were let to fuse under continuous magnetic stimulation for 7 more days, and their progression was monitored using both microscope and stereoscope imaging (Figure S5, Supporting Information). Figure 2h shows the top and side views of MCAs loaded with 30 µg and 60 µg of Fe_3_O_4_ and stimulated after 1 week in culture. The images reveal significant effects of MNP loading and magnetic stimulation on the shape and morphology of the implants. The 30 µg Fe condition resulted in a dome‐shaped assembloid, while the higher 60 µg Fe loading produced a more compact, dense tissue structure. This observation indicates that besides rapid assembly there are also slower kinetics during this 4D biofabrication process affecting assembloid shape over time.

### 2.3 In Vitro Evaluation of MCAs

**Figure 3:**
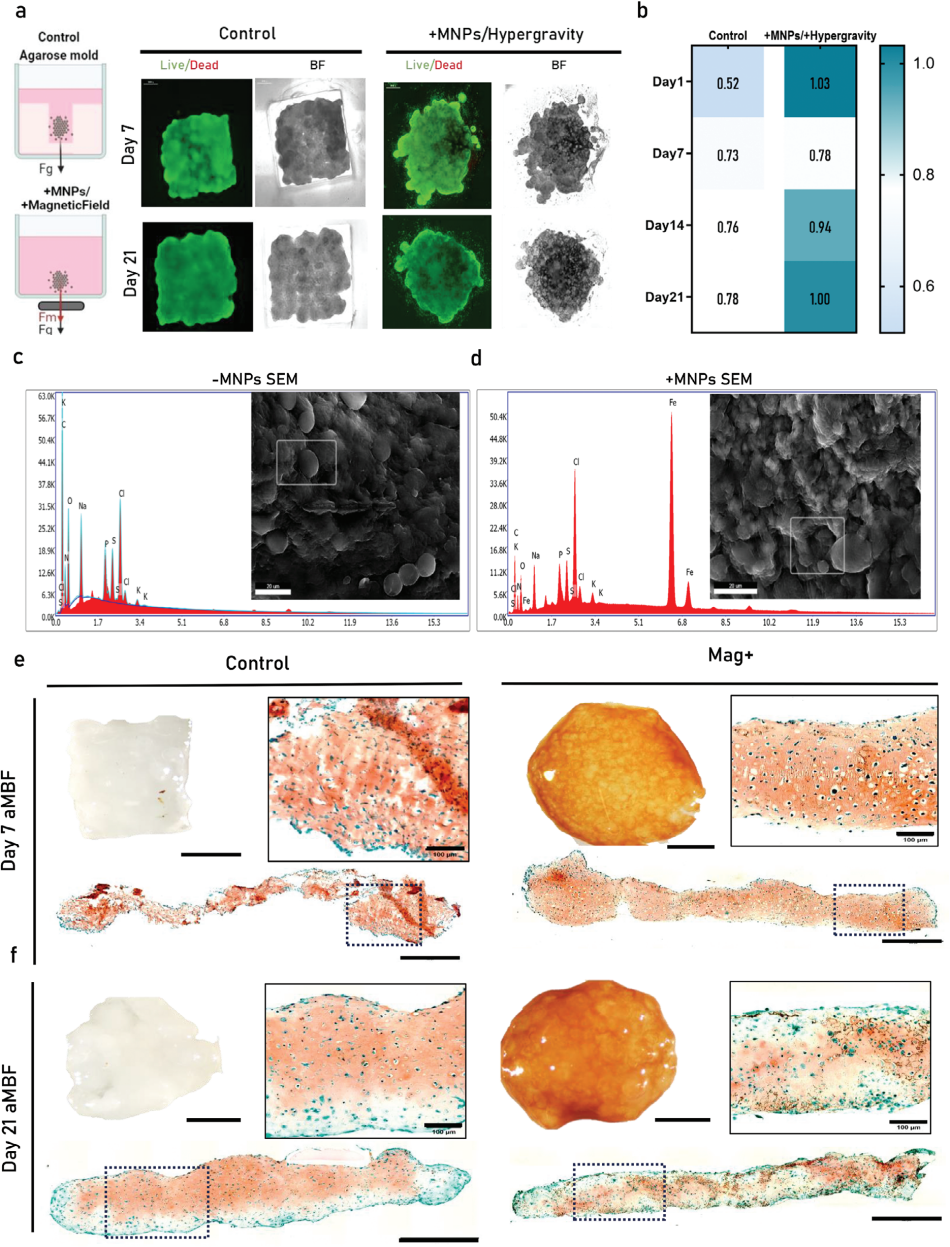
In vitro evaluation of magnetic biofabricated and stimulated cartilaginous assembloids. a) Schematic illustration of our two comparison groups, the control non stimulated group and the magnetic biofabricated and stimulated group (Mag+) and the analysis that were evaluated over time of in vitro maturation along with Live/Dead microscope images showing the assembloids in different timepoints of maturation. b) Heatmap showing the tissue area over mold/magnet surface area (average value, *N* = 3). c) SEM image and elemental diffraction spectra EDX analysis for control and d) Mag+ day 21 assembloids. e) SafO staining on tissue sections obtained from samples on Day 7 and f) Late Day 21 timepoint.

After validating our computational model and assessing the influence of system parameters on biofabrication outcomes, we proceeded with a new series of experiments using 30 µg of Fe per mL due to its reduced cytotoxicity. In these experiments we induced chondrogenic differentiation of the assembled tissues and evaluated their properties at an early day7 and day14 after microtissue fusion. In addition we included a control condition where non‐MNPs loaded microtissues self‐assembled in a restricted non‐adherent well to restrict interaction volume,^[^ ^32^ ^]^ (**Figure** **3**a). Live/Dead imaging in Figure 3a demonstrated a minimal presence of dead cells for both differentiation time points between the magnetically stimulated and control conditions. In Figure 3b, the heatmap illustrates the tight MCA size control obtained by the Mag+ condition relative to the magnet's surface area. A summary of the data extracted from image processing methods regarding their size and growth over time is presented in Figure S6, Supporting Information. MNPs were observed to be present within the ECM components of the MCAs, by employing SEM, we captured images and conducted elemental analysis on the composition of in vitro assembloid tissues on Day 21. We were able to highlight distinct elemental spectra indicating iron peaks specifically in the Mag+ group obtained from cross‐sectional samples (Figure 3c, control) and (Figure 3d, Mag+). MCAs were able to maintain their magnetic actuation properties throughout the in vitro maturation process, in Video S3, Supporting Information Day 21 MCAs can be seen moving under the influence of an external static magnetic field. Interestingly, our results revealed morphological and phenotypical distinctions between the two groups upon staining with SafO on Day 7 (Figure 3e) and on Day 21 (Figure 3f). At the early time point (day 7), the control group showed less maturation in terms of both hypertrophic cell morphology and ECM composition compared to the Mag+ group. However, by the late timepoint, both groups had developed into the hypertrophic chondrocyte cell phenotype.

### 2.4 Two‐Photon Microscope Analysis of the Assembloids

**Figure 4:**
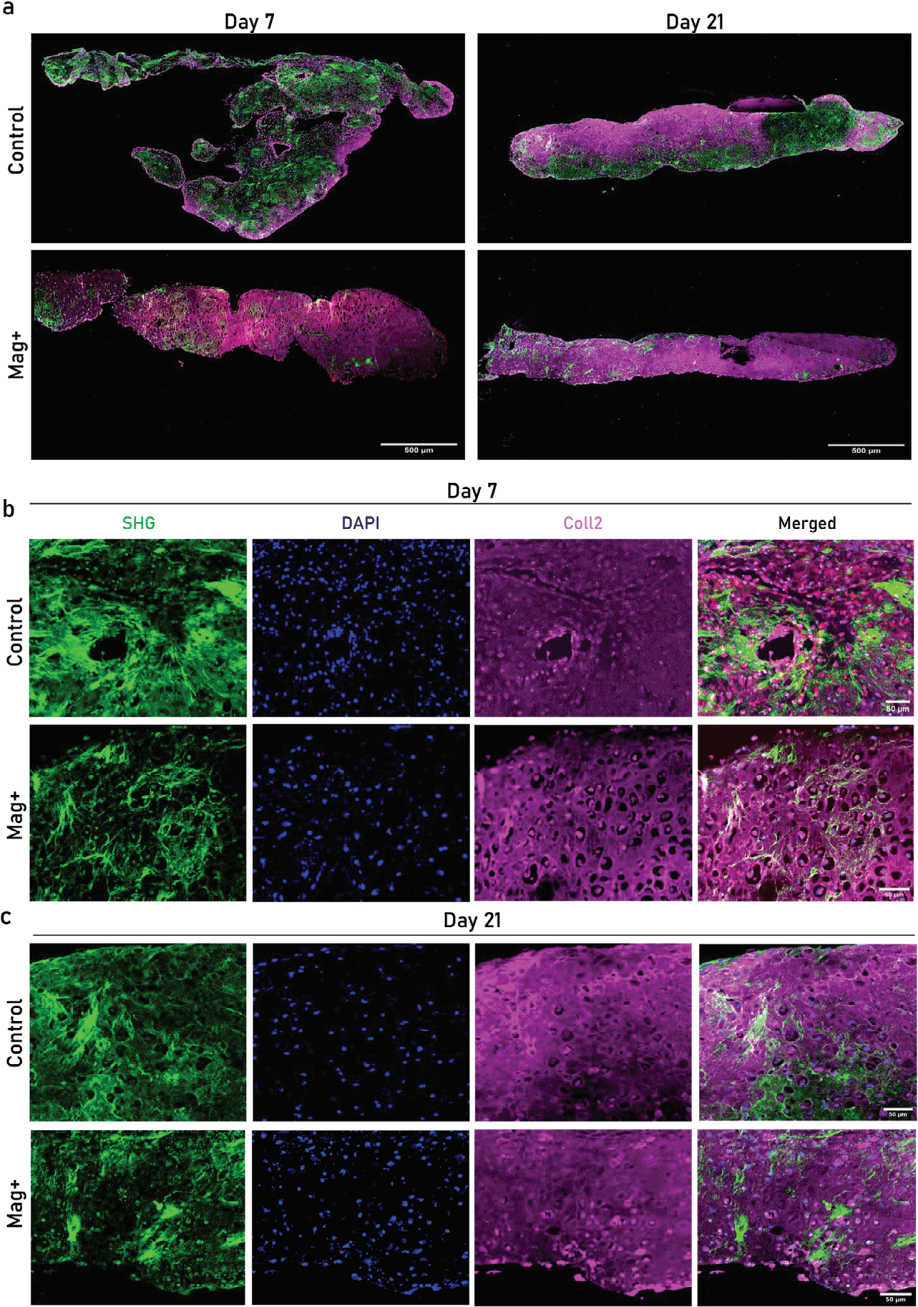
Two‐photon microscope analysis on in vitro assembloids. a) Two‐photon images of both Control and Mag+ Day 7 and b) Day 21 assembloids showing in green (SHG), in blue (DAPI), in Magenta (COL2), in red (OSX) and the merged composite image. Scalebar: 500 µm. c) Two‐photon images of both Control and Mag+ whole assembloids on Day 7 & 21, showing in green (SHG), in blue (DAPI), in Magenta (COL2), in red (OSX) and the merged composite image. Scalebar: 50 µm.

Samples on Day 7 and Day 21 MCAs and their corresponding non‐stimulated controls were visualized by staining for human collagen Type II (COL2) with cell nuclei counterstained using DAPI. Moreover, highly organized collagen fibers were visualized via second harmonic generation (SHG) imaging using two‐photon microscopy, capturing the forward‐scattered light to reveal the collagen's highly ordered structure (**Figure** **4**a). Differences in cellular morphology and matrix organization were evident based on the distinct expression and localization patterns of COL2. In Figure 4b, zoomed images of Day 7 samples highlight the distinct tissue compartments achieved between the two conditions. On Day 7, the SHG signal in the control group was broadly distributed across the sample, while few COL2‐positive areas were localized primarily paracellularly and at the periphery of the control condition assembloids. In contrast, in the Mag+ condition more pronounced COL2 staining was obtained while chondrocyte like lacunae were detected throughout the sample while lower presence of SHG was observed. By Day 21 (Figure 4c), both groups exhibited positive SHG and COL2 signals, though the control sample still showed ECM regions that were negative for COL2. These findings suggest differential matrix organization and collagen deposition dynamics between the groups, particularly influenced by the presence of magnetic stimulation in the Mag+ condition.

### 2.5 Fiber Orientation and Patterning in Assembloids

**Figure 5:**
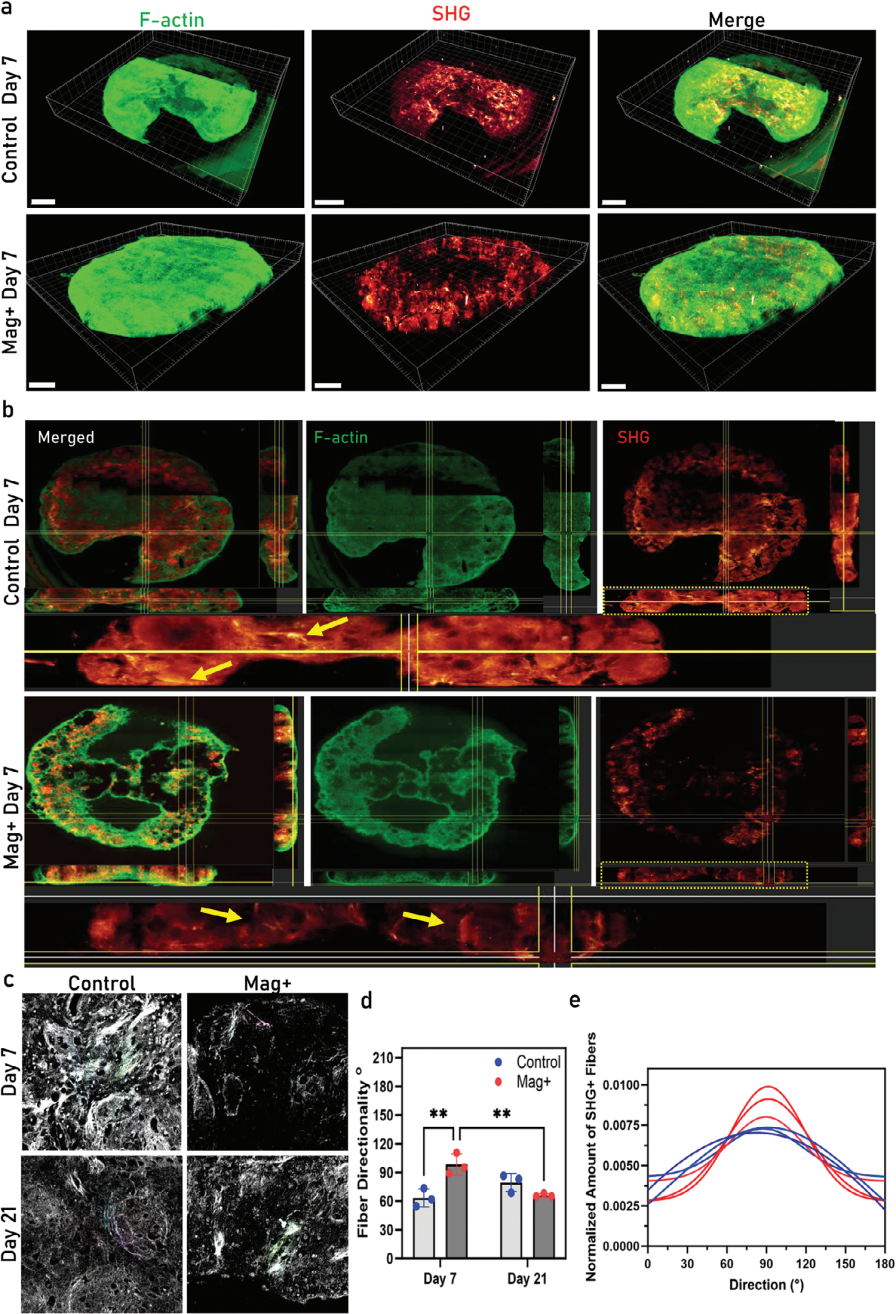
Fiber orientation and directionality analysis. a) 3D reconstructed two‐photon microscope images of whole mount assembloid samples on Day 7 visualized for F‐actin (green), SHG (red) and the merged composite image. b) XY. YZ and XZ directional planes used to showcase the differences in tissue morphology and fiber orientation on Day 7. c) Fiber orientation images from cross sections of assembloids on Day 7 and Day 21 used for measuring the fiber direction. d) Average Fiber direction angles(^o^) measured from 3 different sample regions (N = 3). e) Gaussian distribution of the normalized number of fibers and their corresponding angles for 3 individual replicates of Day 7 samples for both conditions. **p < 0.01; two‐way analysis of variance (ANOVA) followed by Tukey's multiple comparison test. Scalebar:500 µm.

In parallel, we analyzed whole mount assembloids on Day 7 for both conditions. We stained the samples with phalloidin to visualize F‐actin and generated SHG signals to visualize the crystalline structure of collagen fibers, as previously described. In **Figure** **5**a, the 3D reconstructed images of F‐actin and SHG, along with their merged composite images, revealed distinct fiber patterning for each condition. Detailed analysis of the images in the xy, yz, and yx planes (Figure 5b) showed different fiber orientations between the two conditions. The SHG signal in the control condition appeared more abundant with a more horizontal fiber alignment (yellow arrows), whereas in the Mag+ condition, the fibers were vertically aligned across the entire assembloid. Cross‐sections of the samples were later used to analyze fiber directionality. Samples from both conditions and time points were analyzed after image processing with Fourier transformation functions. The average fiber directionality was calculated based on the distribution of fibers and their corresponding angles. As shown in Figure 5d, the fibers in the control condition exhibited a more random distribution, indicated by the broader valley in the Gaussian distribution plot (Figure 5e), while in the Mag+ condition, the fibers were aligned with an average angle of 91° on Day 7 compared to 62° for the control. In the late time point samples, the fiber direction in the control condition aligned with the previous observations for the Mag+ condition, while an opposite trend was observed for the Mag+ condition. These observations suggest that the magnetic field, in combination with the exposure to MNPs, promoted the alignment of collagen fibrils along the same axis as the applied magnetic field.

### 2.6 Evaluation of Bone Formation In Vivo

**Figure 6:**
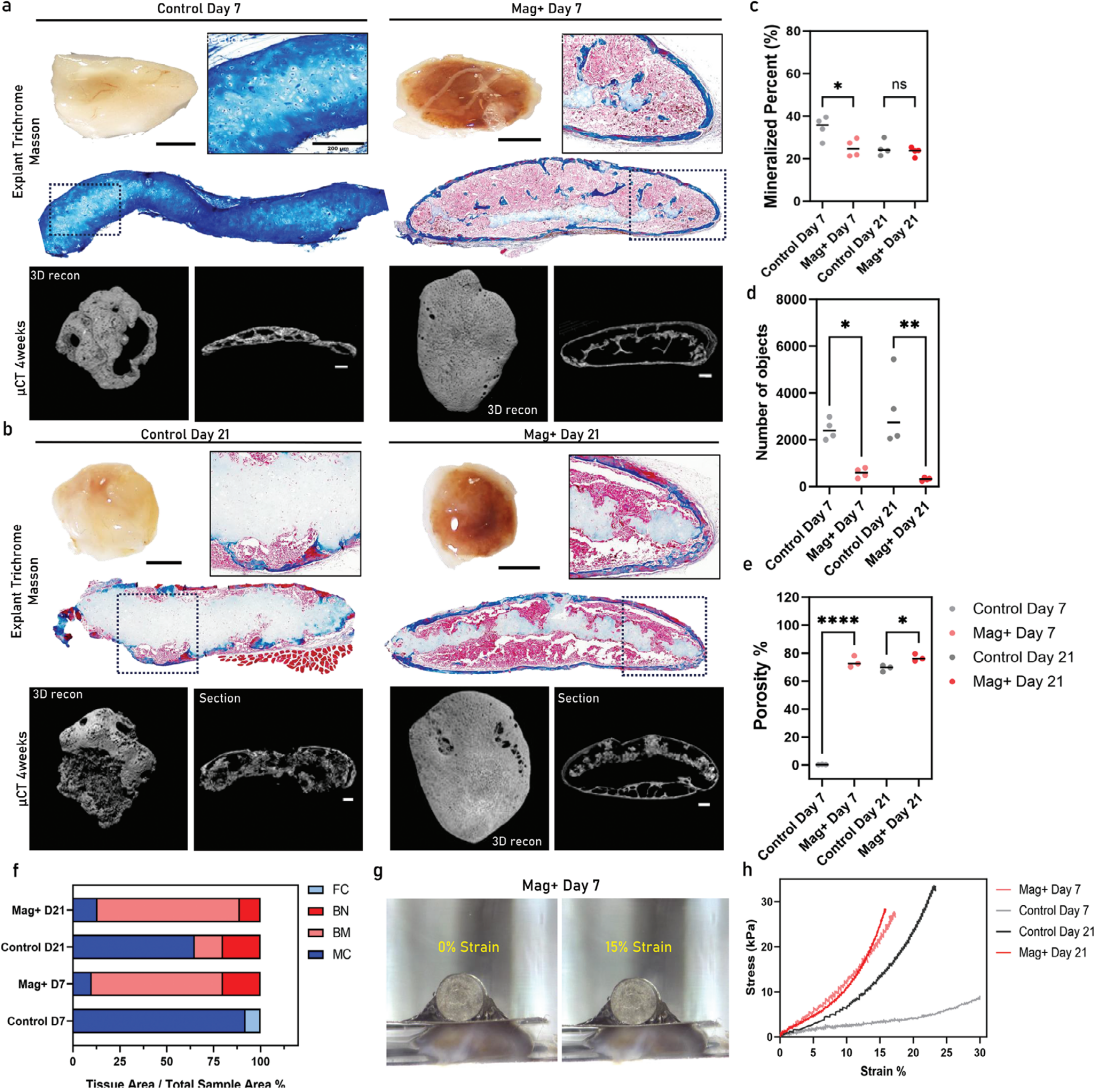
MCAs accelerate bone formation upon in vivo implantation. a) Stereoscopic images of the explants of Day 7 assembloids of both control and Mag+ condition after 4 weeks ectopically in vivo. Trichrome Masson's staining was performed on tissue cross sections with a ROI zoomed in image, along with µCT data of the corresponding samples. b) Stereoscopic images of explants from Day 21 assembloids of control and Mag+ condition and their corresponding Trichrome Masson's stains and µCT images. c) Mineralized percentage % plot between the two conditions on Day 7 and Day 21 assembloids (N = 4). d) Average Number of Mineralized objects plot showcasing the maturity and rigidity of the newform bone (N = 4). e) Bone marrow percentage % measured after analyzing the cavity spaces with successful cortical bone formation (N = 4). f) Bone tissue area percentage measurements conducted on trichrome Masson‐stained sections (FC=Fibrotic Cartilage, BN=Bone, BM=Bone Marrow, MC=Mineralized Cartilage, quantification corresponds to the presented histological images in a) and b). g) Compressive mechanical testing of Mag+ Day7 sample, photos obtained on 0% and 15% Strain. h) Stress – Strain plot showcasing the distinct mechanical properties between the conditions. *p < 0.1; **p < 0.01; ***p < 0.001; ****p < 0.0001 one‐way analysis of variance (ANOVA) followed by Tukey's multiple comparison test. Scale bars: a, b 250 µm.

Following the in vitro analysis, in vivo experiments at day 7 and day 21 were performed by subcutaneously implanting MCA and control groups in immunodeficient mouse models. It is important to note that prolonging the in vitro culture period beyond this timeframe has been shown to induce an inert phenotype known as “over‐maturation.” This over‐maturation leads to a diminished regenerative capacity and poor bone formation upon implantation, making it critical to limit the in vitro culture period to prevent this adverse outcome. The aim of this experiment was to explore whether magnetic stimulation affected cartilage to bone transition capacity (endochondral ossification process). Our results demonstrated a clear difference in the outcome for day 7 implants between the two conditions, the negative bone regeneration control can be seen in Figure S7, Supporting Information. The control assembloid explant, after 4 weeks of implantation, demonstrated the presence of only mineralized cartilage as depicted by Trichrome Masson's staining images (**Figure** **6**a). In contrast, the MCA explants were able to undergo a full cartilage to bone transition and develop a full ossicle containing all necessary compartments such as cortical and trabecular bone, bone marrow, while a very small presence of mineralized cartilage was detected at the center of the explant. For day 21 implants, both conditions were able to give rise to ossicles however in the control condition a very large, mineralized cartilage compartment was seen in the histological sections (Figure 6b), as evidenced by both the Trichrome Masson's staining images and µCT reconstructed images. In Figure 6c–e, mineralized percentage was quantified, the average number of mineralized objects and bone marrow percentage, using µCT data and CT analysis tools. We found that the Mag+ condition on day 7 had fewer mineralized compartments than the control explant (Figure 6d). However, it exhibited more interconnected mineralized objects (Figure 6d) and was filled with bone marrow cavities (Figure 6e). In parallel, we analyzed the tissue‐specific area percentage based on Trichrome Masson staining for specific bone areas such as Fibrocartilage (FM), Bone Marrow (BM), Bone (BN), and Mineralized Cartilage (MC). Image processing methods based on color threshold were used for this analysis, and the results are shown in Figure 6f. The Mag+ condition formed cortical bone compartments with rich bone marrow cavities from the early implantation time point and exhibited a similar trend at the later implantation time point. In contrast, the control condition only remodeled and formed bone and bone marrow at the later time point, but with high presence of mineralized cartilage compartments compared to Mag+. The full set of µCT analysis results for all replicates can be seen in Figure S8, Supporting Information. To further highlight the differences observed between the two conditions, we evaluated the mechanical properties of the explants. As shown in Figure 6g, the Mag+ explant is depicted before and after the application of compressive force, with the resulting stress‐strain curves presented in Figure 6h. The Mag+ samples exhibited consistently high mechanical properties across both implantation timepoints, reaching 29 kPa at 15% strain. In contrast, while the control samples achieved comparable mechanical properties at the later timepoint (32 kPa at 20% strain), they displayed significantly poorer mechanical performance at the early timepoint, with only 8 kPa at 30% strain. These findings underscore the enhanced mechanical stability provided by the Mag+ treatment, particularly at earlier stages of implantation. Similarly to the CT data, these findings align with our earlier observations of collagen organization via COL2 staining and SHG imaging. The early distribution of COL2 and SHG signal, in the Mag+ group, reflects enhanced ECM organization and collagen alignment, which likely supported the earlier onset of bone formation observed by the increased mechanical properties.

### 2.7 RNA Sequencing of in Vitro Assembloids Prior Implantations

**Figure 7:**
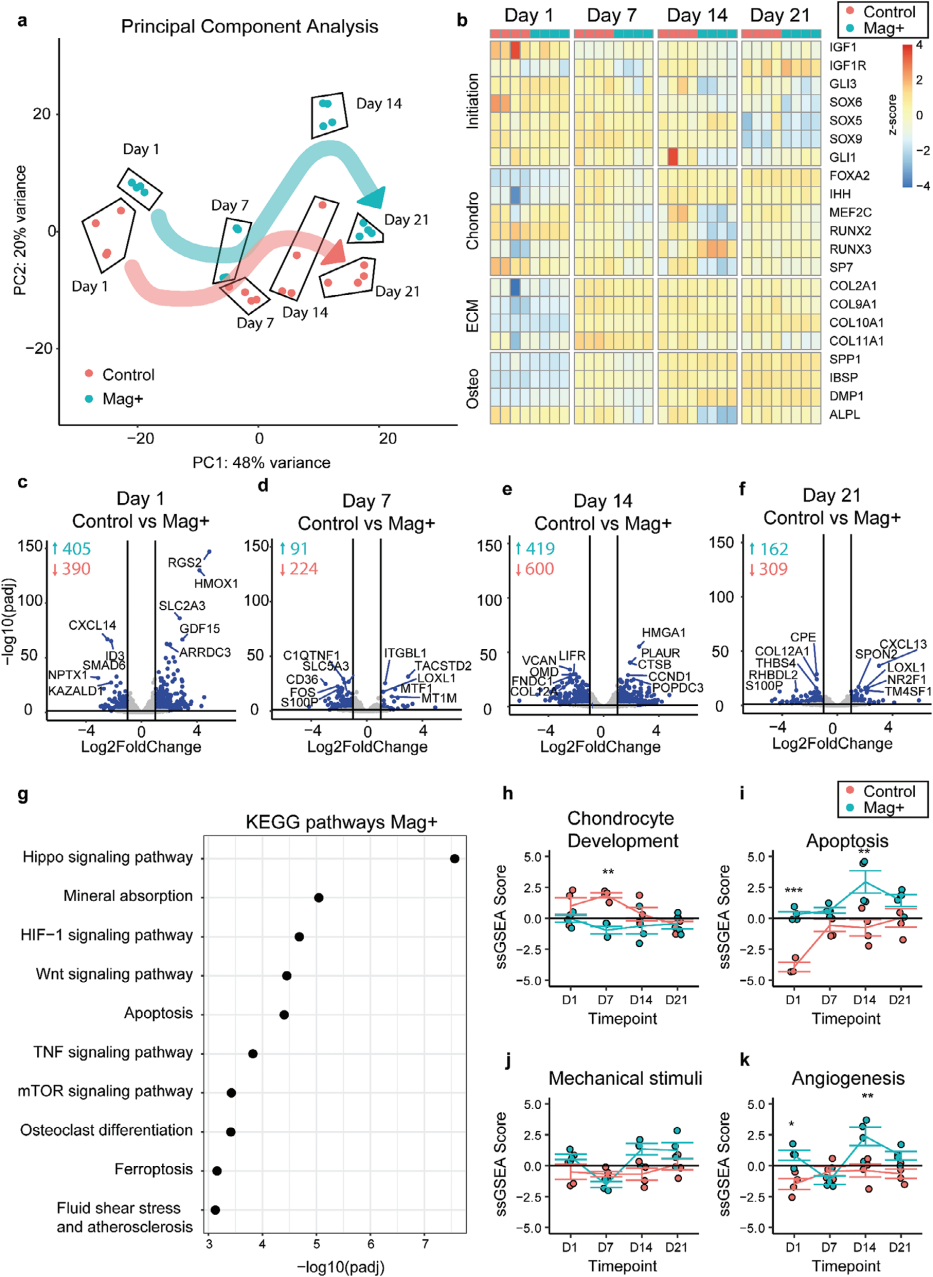
Transcriptome analysis comparing magnetically stimulated versus control over time. a) Principal component analysis (PCA) showing the differentiation trajectories. b) Representation of gene sets commonly linked to critical stages of endochondral ossification. c–f) Differential expression (DEG) analysis between control and magnetically stimulated samples per timepoint, indicating the total amount of DEGs and highlighting the 5 most significant up‐ or downregulated genes per timepoint. g) KEGG pathway enrichment between control and magnetically stimulated samples, excluding time as variable. h–k) single sample gene set enrichment analysis (ssGSEA) of selected gene ontology terms.

Transcriptome analysis was performed to investigate whether magnetic stimulation affected the underlying mechanisms that drive chondrogenic differentiation and induction of hypertrophy as it is a critical determinant for endochondral bone formation. Principal component analysis (PCA) in **Figure** **7**a indicated a clear distinction of timepoints and similar trajectories of samples cultured in Mag+ or control conditions. Figure 7b shows key genes involved in chondrogenic differentiation, extracellular matrix (ECM) production and hypertrophic differentiation. Early genes showed high expression of this gene panel on day 1, followed by a decrease toward day 21. In the Mag+ condition, these genes started to decrease from day 7, while control samples continued to express early markers up to day 14. From day 7, both conditions expressed markers of differentiation, however, on day 14, there was a drop in the Mag+ condition in the expression of osteogenic genes, RUNX2, SP7 and ALPL. Yet by day 21, these markers showed similar expression profiles. Differential expression analysis between the conditions for each timepoint, shown in Figure 7c–f, indicated a 405 and 390 differentially expressed up‐ or downregulated genes (DEG) on day 1 as a result of magnetic stimulation. Over time, the amount of DEGs decreased to 162 upregulated and 309 downregulated genes on day 21. Next, KEGG pathway analysis was performed by comparing Mag+ and control samples, excluding time as variable. Figure 7g shows enriched terms which were up regulated in the Mag+ condition. Terms like Hippo signaling, Wnt signaling, mTOR signaling, and Fluid shear stress highlighted the fact that the effect of mechanical stimulation was seen also at the gene level. Magnetic stimulation also enhanced catabolic pathways such as mineral absorption and osteoclast differentiation. In addition, apoptotic pathways were enriched, especially iron‐induced ferroptosis in combination with its counterpart TNF signaling^9^. Finally, a single sample gene set enrichment analysis (ssGSEA) was performed, assigning an enrichment score per sample per gene ontology term, based on the cumulative expression of its genes^6^. Figure 7h–k shows these scores over time for chondrocyte development (GO:0 002063), apoptosis (GO:00 97190), mechanical stimuli (GO:0 009612), and positive angiogenesis (GO:00 45766). This showed an offset in chondrocyte development on day 7, which stabilized to day 14 and 21. ssGSEA scores for mechanical stimuli were overall significantly increased, but individual timepoints did not reach significance. Interestingly, the Mag+ samples had a consistent higher expression of pro‐angiogenic genes. The main genes of hypertrophic differentiation as well as an extended table of ssGSEA plots can be found in Figure S9, Supporting Information.

### 2.8 MNPs Presence and Quantification for n Vitro and In Vivo Samples

**Figure 8:**
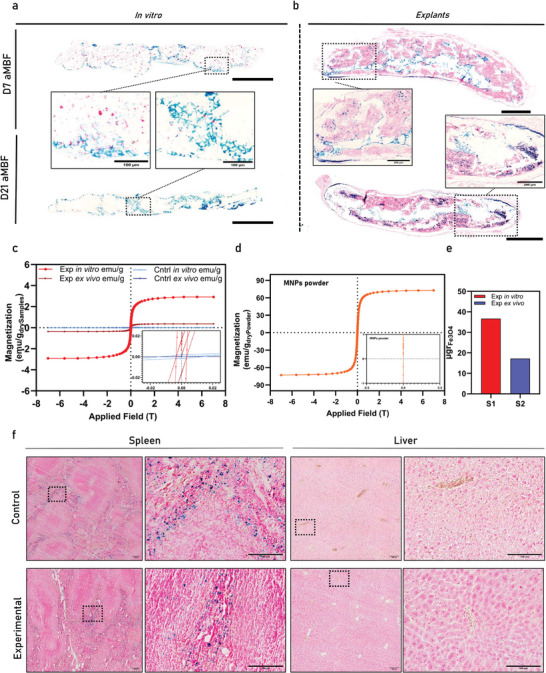
MNPs monitoring through in vitro and in vivo processes. a) Prussian Blue staining verifying the presence and localization of iron depositions found in pericellular honeycomb structures in vitro and b) in the periphery of the mineralized cartilage and inside the bone marrow compartment (explants). c) Magnetic hysteresis curve of magnetic treated in vitro and ex vivo dry samples (emu g^−1^ drySample) showcasing the superparamagnetic behavior offered by the IONPs. d) Hysteresis curve for the bare IONPs (emu g^−1^ dryPowder Fe_3_O_4_) used as a reference to quantify the contribution and amount of Fe_3_O_4_ per sample. e) Iron oxide content (µg) measurement comparison between in vitro and ex vivo samples showing 45% reduction after 4 weeks in vivo. f) Histological evaluation with Prussian blue staining of spleen and liver biopsies obtained from the animals that were used as an ectopic model. Control group corresponds to the animals that were used for implantation of the non IONPs treated assembloids tissues. Scalebars: a) 100 and 200 µm, e) 200 µm.

In this final part of the work, we aimed to evaluate, localize, measure, and track the presence of MNPs during both the in vitro and in vivo bone remodeling process. Cross sections from MCAs were obtained, along with ossicle explants from the corresponding implantation timepoints. **Figure** **8**a showcases the Prussian blue staining results for in vitro MCA for both timepoints. The presence of iron was also found in our ossicles with most of the positive signal detected along the periphery of the mineralized cartilage remnants or within the bone marrow compartments (Figure 8b). This indicates successful bone tissue remodeling, demonstrated by the degradation of cartilaginous ECM compartments upon implantation. To quantify MNP loss during implantation and ossicle formation, samples from both MCA implant ossicle‐explant analyzed with a SQUID magnetometer to measure the exact amount of iron (Fe). In Figure 8c, the characteristic sigmoid spectrum of superparamagnetic nanoparticles is shown for MCAs, while the control samples exhibited flat lines due to the absence of superparamagnetic components. A reference powder of the magnetic nanoparticles (MNPs) used in this study was employed to calculate the microgram quantities of iron (Fe) present in both the in vitro and explant samples, as shown in Figures 8d,e. Specifically, a known mass of the reference powder (0.1 mg of dried MNPs) was used to determine the magnetic moment in emu g^−1^ of Fe. This value was then applied to convert the magnetization data obtained from the SQUID measurements to the amount of iron (in micrograms) in the experimental samples. The calibration derived from the reference dried powder of known mass provided an accurate quantification of the Fe content and its magnetization mass (emu g^−1^), which facilitated the comparison of the magnetic properties across different sample groups. In this way, we were able to accurately measure the quantity of Fe in our samples, and by subtracting the µg percentage, we report a 53% reduction in the amount of MNPs measured^[^ ^47^ ^]^ between the implants (in vitro day 21 MCA, 36.7 µg Fe_3_O_4_) and explants after 4 weeks (17.2 µg Fe_3_O_4_) indicating their removal from the implants as a result of vessel infiltration and bone marrow formation. To ensure that the use of MNPs and our process did not negatively affect mouse physiology, we isolated and performed necropsies on both spleen and liver organs. These were stained with Prussian blue to observe any abnormalities. As shown in Figure 8f, both organs appeared physiological under microscopic evaluation, with no excess accumulation of iron deposition reported in these organs at the time of sacrifice.

## 3 Discussion

Growing literature around magnetically‐aided tissue engineering is reporting that microstructural cues and biomolecule guidance act in tandem to direct cell fate commitment in bone,^[^ ^48^ ^]^ tendon,^[^ ^49^ ^]^ cartilage.^[^ ^50^, ^51^ ^]^ This ever‐increasing sophistication of magnetically aided tissue engineered constructs should be further explored and validated with functional in vivo outcomes. In this study, we explored 4D magnetically driven biofabrication of cartilaginous microtissue‐based assembloids^[^ ^30^ ^]^ using MNPs. We studied the magnetically driven assembly process as well as the influence on the emergent assembloid tissue properties. Subsequently the modulation of the differentiation processes was mapped and finally validated in vivo demonstrating the enhanced bone functionality of the magnetically augmented MCAs. Critical size bone defects constitute an unmet clinical challenge which often results in non‐unions due to the fact that its size exceeds the intrinsic capacity of self‐regeneration and consequently bone repair is delayed and impaired.^[^ ^52^ ^]^ Cartilaginous microtissues have been shown to be able to recapitulate innate processes encountered in fracture healing and mimic to a certain extent the properties of the fracture callus demonstrating robust cartilage to bone transition.^[^ ^32^, ^38^ ^]^ Hence, magnetically augmented implants possessing superior bone forming capacity due to their remote stimulation, as investigated in this work, can contribute to mitigating this challenge.

High‐density tissue biofabrication through the use of microtissue building blocks is gaining traction through the use of novel technologies such aspiration assisted biofabrication^[^ ^22^, ^23^, ^25^ ^]^ or laser‐assisted biofabrication and also recently demonstrated for extrusion bioprinting.^[^ ^53^ ^]^ However, these approaches do not provide the capacity to affect slower emergent biological processes such the formation of extracellular matrix networks and definition of phenotype. The development of magnetic biofabrication using microtissues^[^ ^54^, ^55^ ^]^ in junction to low toxicity super paramagnetic iron nanoparticles^[^ ^56^ ^]^ opens novel capabilities in guiding tissue properties in junction to biological processes of the formed tissues. As shown in Figure 1, we conducted cytotoxicity assays to determine MNP concentrations that maintain cell viability and functionality while enabling magnetic control. Iron concentrations above 60 µg mL^−1^ hindered cellular proliferation, while lower levels were well‐tolerated, highlighting the importance of fine‐tuning dosage. At these concentrations, magnetized microtissues responded effectively to static magnetic fields, promoting rapid and efficient bioassembly. Given the possibility to generate complex‐shaped magnetic fields when controlling magnet shape, intensity and inhomogeneity, field orientation, time dependence and/or concentration of MNPs, computational design tools to provide a view of the biofabricated tissue implant, become indispensable. In this work, we developed an agent‐based model^[^ ^57^ ^]^ in order to predict MCA fusion dynamics and final assembloid shape similar approach has been followed in magnetic levitation experiments for articular chondrospheres,^[^ ^13^ ^]^ as shown in Figure 2. Model predictions matched well with experimental observation rendering this tool useful for design of more advanced microtissue‐based assembloids in the future. In our study we coupled the original model information and we tried to quantify the mechanical stimulation exerted on the microtissue collective during the magnetic driven assembly process, as well as the distribution and propagation of mechanical stresses within such structurally heterogeneous assembloid structures.^[^ ^58^ ^]^ The model could provide information on all vectors of the generated stresses capturing both hypergravity and radial stresses. Interestingly in our simulations we see the formation of a dome shape tissue, something that was also experimentally confirmed qualitatively through stereoscope imaging. We should note that cartilaginous microtissues are highly complex structures and more detailed mathematical models capturing cell and ECM compartments would in the future be required for the precise mechanical cues that cells experience. Nevertheless, the present tool can be used for exploration of different magnetic field strengths and configurations to further enhance tissue assembly and functionality. During biofabrication and especially post‐biofabrication mechanical stresses, mostly due to compaction, will be generated. These were quantified in a volume averaged manner per microtissues module through the mathematical model. Cartilaginous cells known to be mechanoresponsive in previous studies cells/microtissues were exposed to stresses of 400–200 kPa^[^ ^59^ ^]^ which compared to those encountered in our study. Further understanding mechanical loads and their remote‐control post implantation will be critical for successful outcomes as recent studies have demonstrated the importance of controlled loading for long bone fracture healing.^[^ ^38^, ^60^, ^61^ ^]^ Controlling this further and including dynamic magneto‐mechanical stimulation which has been seen to enhance mechanical properties of tissue engineered cartilaginous tissue^[^ ^62^ ^]^ could enable to even mimic gaiting and could be further explored in future studies focusing in orthotopic in vivo experiments.

In vitro experiments showed that after 4 weeks MNPs were still present within the MCAs (Figure 3) and that magnetic stimulation during chondrogenic differentiation accelerated differentiation kinetics as evidenced by the presence of chondrocytes cells at the early time point with a more prominent presence of Safranin O (Figure 3c) which reflect the presence of sulfated glycosaminoglycans, a key component of cartilage ECM. Similar observations have been seen for mechanically stimulated tissue engineered cartilaginous constructs.^[^ ^63^, ^64^, ^65^, ^66^ ^]^ This was further corroborated by the earlier and pronounced expression and localization of collagen Type II and Osterix (Figure 4). Experimental observations using whole assembloid image analysis (light sheet microscopy, Figure 5) revealed distinct fiber orientation for the early time point, with magnetic conditions promoting a more vertical alignment of collagen fibers as compared to control. These findings suggest that magnetic stimulation not only aids in microtissue assembly but also influences the microstructural organization of tissue ECM.^[^ ^67^ ^]^ ECM alignment is crucial both for their mechanical properties^[^ ^65^ ^]^ as well as functionality in regards to cartilage to bone transition as it has been linked to the recruitment of progenitor cells and host vessels.^[^ ^68^ ^]^ Interestingly ectopic implantation studies further demonstrated the enhanced bone formation capacity of magnetically stimulated assembloids compared to controls for both time points assessed (Figure 6). The most striking difference was observed in the early timepoint where Mag+ explants were able to develop full ossicle with clear cortical, trabecular and bone marrow compartments being formed as opposed to the control condition where only mineralized cartilage was generated. For the more mature assembloid implants we still observed striking differences concerning the amount of bone marrow formed within Mag+ and control conditions. It is interesting to note that for the Mag+ condition explants exhibited a reproducible dome shape (Figure 2i) indicating a maintenance of the original implant shape and dimensions. Taken together magnetic 4D biofabrication holds promise to create more potent and effective bone forming grafts which could result in superior bone defect regeneration outcomes; however, this should be further proven in future studies through implantation in an orthotopic tibial in vivo model. Additionally, engineering cartilaginous microtissues used for tissue engineered implants, destined for skeletal defect regeneration will be exposed to mechanical stresses during and post implantation. This applies for both long bone defects as well as osteochondral defects of the knee joint. In both cases a transition from the implanted immature cartilage to hypertrophic cartilage leading to mineralization and bone formation will be processes that these microtissues will need to undergo.

To decipher the molecular mechanisms behind these intriguing in vivo results we carried out transcriptomics analysis to investigate whether magnetic stimulation affected chondrogenic differentiation and whether this could explain these distinct in vivo outcomes (Figure 7). Principal component analysis (PCA) indicated clear distinctions between timepoints and similar trajectories for samples cultured in Mag+ or control conditions in terms of chondrogenic differentiation and commitment to pre‐hypertrophic phenotype was obtained as for example demonstrated by single microtissue studies.^[^ ^32^ ^]^ Sample gene set enrichment analysis (ssGSEA) showed that chondrocyte development stabilized from day 7 to day 21 (Figure 7b). Key genes involved in chondrogenic initiation (IGF1, SOX9, GLI1), differentiation (IHH, SP7, RUNX2), ECM production (COL2A1, COL9A1, COL10A1), and hypertrophic/osteogenic differentiation (IBSP, SPP1, DMP1) were upregulated.^[^ ^69^ ^]^ KEGG pathway analysis indicated enriched terms upregulated in the Mag+ condition, such as Hippo signaling Fluid shear stress, highlighting the effect of mechanical stimulation. Hippo/Yap signaling is involved in the process of regulating mechanotransduction.^[^ ^70^ ^]^ In fracture healing hippo signaling has been recently linked to improved bone formation in the cartilaginous fracture callus mediated by WNT signaling (also observed as one of the most upregulated pathways in our data set,^[^ ^71^ ^]^ while additional information reveal the link between Tenascin mediated activation and hippo signaling to enhancement of chondrogenic differentiation in vitro and cartilage to bone transition in mouse models^[^ ^72^ ^]^ indicating its link to functional outcomes. In the context of cartilage to bone transition in osteoarthritis YAP overexpression has been seen to be activated with compression^[^ ^73^ ^]^ and resulted in increased expression of inflammation and catabolic process linked to matrix remodeling, as seen also in our study, while suppression of YAP inhibited catabolic genes expression and chondrocytes apoptosis.^[^ ^74^ ^]^ Wnt pathway is also an additional pathway that has been seen to play role in controlling cell fate and differentiation of skeletal cells under the influence of mechanical signals especially seen in non‐canonical Wnt/Ca+ signaling.^[^ ^13^, ^75^ ^]^ In the context of OA it has been seen that strong activation of the canonical Wnt signaling pathway alters the molecular characteristics of healthy articular chondrocytes driving their further differentiation toward hypertrophic cells associated with the production of a calcified ECM.^[^ ^14^, ^15^ ^]^ In the context of OA it has been shown that strong activation of the canonical Wnt signaling pathway alters the molecular characteristics of healthy articular chondrocytes driving their further differentiation toward hypertrophic cells associated with the production of a calcified ECM.^[^ ^76^, ^77^ ^]^ In addition, WNT signaling was recently seen to play a critical role in long bone fracture healing mediating cartilage hypertrophy and transition into bone.^[^ ^78^ ^]^ Mechanosensitive ion channels, such as stretch‐activated channels, contribute to cellular mechanotransduction by converting mechanical forces into electrochemical signals, thereby influencing gene expression, and differentiation^[^ ^79^ ^]^ and this pathway was differentially expressed in our magnetically stimulated control. In addition, mechanical force‐activated calcium signaling pathway could act in synergy with Wnt signaling. Mechanosensitive ion channels Piezo1/2 and transient receptor potential vanilloid 4 (TRPV4) are two key calcium channels that respond to mechanical forces, and recent investigations have illustrated that Wnt signaling pathway can interfere with their mediated regulation of bone metabolism.^[^ ^80^ ^]^ However, in order to establish clear interaction between Piezo1 or TRPV4 channels and the Wnt pathway in cartilage to bone transition systems such as in OA or cartilaginous engineered implants such as the one developed in the present study, additional dedicated research is needed. Hence, Hippo signaling can act as a robust promoter of tissue regeneration, underscoring the potential of targeting the Hippo pathway in regenerative medicine^[^ ^81^ ^]^ while our study indicates this pathway as a target pathway for future magnetic stimulation studies.

Accelerated mineral transport and enriched apoptotic pathways were also observed and is something expected for hypertrophic chondrocytes in order to form bone.^[^ ^82^ ^]^ This might be linked also particularly to ferroptosis a recently identified type of programmed cell death connected to iron accumulation^[^ ^83^ ^]^ and has been observed to play a role in cartilage to bone transition in osteoarthritis^[^ ^84^, ^85^ ^]^ linked to upregulation of MMP13 and acceleration of ECM remodeling (degradation).^[^ ^86^, ^87^ ^]^ Nevertheless, iron accumulation and its role in fracture healing and long bone development is still not known. MNPs have been widely investigated for their biocompatibility and safety in various biomedical applications.^[^ ^88^, ^89^ ^]^ Iron oxide nanoparticles (Fe_3_O_4_), as used in this study, are well‐recognized for their biocompatibility and biodegradability. However, their long‐term accumulation, particularly in non‐target tissues, poses potential risks such as oxidative stress, inflammation, or organ‐specific toxicity.^[^ ^90^, ^91^ ^]^ These effects are influenced by multiple factors, including particle size, surface coating, dose, and administration route. In our study, we addressed these concerns by conducting comprehensive evaluations, including MTT, LDH, and ROS assays, as well as examining iron accumulation in key organs such as the spleen and liver. At the timepoints assessed, no cytotoxic effects or adverse phenotypes were observed at either the cellular level or in the host (mice), underscoring the biocompatibility of the MNPs within the tested parameters. To mitigate long‐term toxicity, strategies such as surface functionalization to enhance clearance, optimizing particle size for renal or hepatobiliary excretion, and maintaining appropriate dosing are essential. Additionally, the capacity of cells and tissues to metabolize and utilize iron from MNPs can further minimize potential side effects. This capability also opens pathways for versatile applications where programmable biological functions can be modulated or enhanced through direct or indirect MNP properties.^[^ ^92^, ^93^, ^94^ ^]^

Finally, Mag+ samples consistently showed higher expression of pro‐angiogenic genes which could additionally explain the in vivo results. Taken together it appears that MCAs possessed phenotype promoting increased cartilage to bone turnover with activated catabolic process resulting in the rapid formation of ossicles as seen in our in vivo experiments. Interestingly, a reduction in iron presence was observed in MCA explants over time, indicating successful removal of MNPs from the explants which could be explained by vessel invasion and gradual bone marrow compartment formation (Figure 8). This property could be harnessed for the non‐invasive monitoring of the bone remodeling process through the use of MRI^[^ ^95^ ^]^ providing in the future insights on the kinetics of cartilage to bone transition and fracture healing. Future investigations should focus on systemic immune responses, long‐term biodistribution, and clearance mechanisms using advanced techniques such as sensitive MRI‐based imaging. These studies could provide deeper insight into the behavior of biohybrid magnetic implants and the absorption and destination of MNPs within in vivo systems, further informing their safe and effective use in clinical applications.

Future research could explore the interaction of magnetically augmented microtissue populations with more advanced magnetic setups, such as electromagnetic configurations for direct delivery to defect sites^[^ ^96^ ^]^ and in situ biofabrication of assembloids or permanent magnet configurations used for guiding living within living tissue,^[^ ^97^ ^]^ combined with real‐time magnetic particle imaging. These magnetically augmented microtissues and subsequently formed assembloids can be viewed as multi‐scale programmable tissue structures with the ability to program microtissue motion, final assembloid shape and activation of signaling pathways linked to the mechanism of endochondral ossification and hence the in vivo outcome. This provides a unique category of living tissue robots^[^ ^98^ ^]^ able to perform programmable tasks^[^ ^99^ ^]^ not only in vitro, that is, partial control of differentiation processes, but also post – implantation, that is, ossicle formation. Furthermore we could envisage the use of such living‐tissue‐robots for future non‐invasive magnetic surgery techniques,^[^ ^100^ ^]^ making use of injectable MCAs. In our study, we evaluated the magnetic properties of both in vitro implants and explants after 4 weeks using SQUID magnetometry. The results demonstrated that the MNPs were retained within the tissues, as evidenced by their preserved magnetic properties. This retention was further supported by the ability of the tissues to be externally magnetized and magnetically manipulated. Based on these findings, we are confident that the magnetic properties of SPION‐treated tissues remain functional for at least 4 weeks, suggesting their potential for sustained external actuation, stimulation, or fixation. Further studies will be needed to investigate the long‐term retention and functionality of SPIONs beyond this timeframe to strengthen their clinical applicability. This approach could enable the precise in situ placement, fixation, and post‐operative stimulation of assembloid tissues enhancing the clinical outcome in cases of challenging defects and paving the way for less invasive surgical techniques.

## 4 Conclusion

Our study successfully demonstrated that magnetically augmented cartilaginous microtissue populations can be used for as – designed magnetic biofabrication of functional assembloid implants. The use of magnetic fields affected chondrogenic differentiation cascades at the gene but also ECM level. Magnetically stimulated assembloids exhibited superior bone forming capacity leading to the successful formation of full ossicles in vivo even at time points where control conditions were unable to do so indicating their criticality in guiding bone formation. Transcriptome analysis revealed that magnetic stimulation influences key mechanoresponsive pathway mediating endochondral ossification that could be further targeted in future studies, further underscoring the potential of this approach. This study demonstrates the application of 4D magnetic biofabrication toward the generation of programmable skeletal assembloid implants with remote control, holding great promise for regeneration of challenging bone defects in the future.

## 5 Experimental Section

### MNPs Synthesis and Characterization

Ferrous and ferric chlorides, in a 1:2 molar ratio of FeCl₂·4H₂O to FeCl₃·6H₂O, were dissolved in deionized (DI) water (resistivity ≈18 MΩ·cm) to prepare a 0.1 M iron precursor solution with a pH of 1.8. The precursor solution was freshly prepared before each synthesis. A 0.57 M NaOH solution, prepared from a 2 M NaOH stock solution and DI water, served as the base solution. For growth quenching and stabilization, a 0.32 M citric acid (CA) solution, with a pH of 1.8, was employed. The synthesis of the iron oxide based MNPs required a three‐step synthesis process taking place in a flow reactor as previously demonstrated.^[^ ^46^ ^]^ Shortly, in the first step, base and precursor solutions were mixed rapidly in a T‐mixer using a syringe pump to initiate the reaction. At the second step, the addition of CA solution at a specific time quenched particle growth and permitted the formation of a CA coating layer to prevent particle clumping and stabilize the nanoparticles, which were observed to be fairly stable with an average 6 nm diameter as shown in Figure 1c. Supplementary TEM images with lower particle concentration as well as a DLS measurement on the average hydrodynamic diameter are also provided at Figures S1 and S2b, Supporting Information, respectively. The ζ‐potential of the CA‐coated NPs is −39 mV (Figure S2a, Supporting Information), providing additional evidence for particle stability. Last, in the third step, after adding the CA, IONP solution passed through a coiled flow inverter (coiled PTFE tubing with flow inversions every four turns) for aging with a total residence time of 160 s before collection. Purification of the samples was conducted utilizing the dialysis method. Specifically, 3 mL of as‐prepared IONPs were enclosed within a Thermo Scientific Slide‐A‐Lyzer 10K MWCO G2 Dialysis Cassette. The cassette was kept in a 5000 mL glass beaker containing 4500 mL of DI water and incubated under shaking conditions for 72 h with nine changes of water. To characterize the synthesized magnetic nanoparticles (MNPs), transmission electron microscope (TEM) images were obtained using a JEOL 1200 EX microscope operating at 120 kV acceleration voltage. TEM sample preparation involved placing 20 µl of washed MNP solution onto carbon‐coated copper grids, which were left to dry overnight. The average particle diameter was calculated by measuring at least 100 particles using ImageJ software. The ζ‐potential and dynamic light scattering (DLS) hydrodynamic size of the citric acid (CA)‐coated nanoparticles were determined using a Malvern Zetasizer Nano‐ZS. X‐ray diffraction (XRD) patterns of dry samples were recorded with a PANalytical X'Pert3 diffractometer (Malvern Instruments), using Co Kα radiation (λ = 0.179 nm) and operated at 40 mA and 40 kV.

### hPDCs 2D Expansion

hPDCs were isolated from periosteal biopsies from different donors and were used as cell pools as previously described.^[^ ^101^ ^]^ The hPDC pools were expanded until passage 7 at 37 °C, 5% CO_2_, and 95% humidity in Dulbecco's modified Eagle medium (DMEM, Life Technologies, UK) with 10% fetal bovine serum (HyClone FBS, Thermo Scientific, USA), 1% antibiotic‐antimycotic (100 units mL^−1^ penicillin, 100 mg mL^−1^ streptomycin, and 0.25 mg mL^−1^ amphotericin B), and 1 × 10^−3^ m sodium pyruvate (Life Technologies, UK) and subsequently were used for experiments (in vitro, in vivo, RNA‐seq). All patients provided informed consent, and all procedures were approved (ML7861) by the ethical committee for Human Medical Research (KU Leuven).

### Generation of Magnetic 3D Callus Microtissues

A microwell platform (AggreWell400, STEMCELL Technologies Inc, Canada) was coated with anti‐adherence rinsing solution (STEMCELL Technologies Inc, Canada) following manufacturer recommendations. After 2D expansion for seven passages, hPDCs were washed with PBS and harvested using TrypLE Express (Life Technologies, UK) and were seeded with a cell density 300 000 cells well^−1^, leading to 250 cells microtissue^−1^. Following microwell seeding, cells through self‐aggregation formed the 3D microtissues and were allowed to differentiate in this set up for 7 days using a chemically defined chondrogenic media (C8) composed of: LG‐DMEM (Gibco) supplemented with 1% antibiotic‐antimycotic (100 units mL^−1^ penicillin, 100 mg mL^−1^ streptomycin and 0.25 mg mL^−1^ amphotericin B), 1 mM ascorbate‐2 phosphate, 100 nM dexamethasone, 40 µg mL^−1^ proline, 20 µM of Rho‐kinase inhibitor Y27632 (Axon Medchem), ITS + Premix Universal Culture Supplement (Corning) (including 6.25 µg mL^−1^ insulin, 6.25 µg mL^−1^ transferrin, 6.25 µg mL^−1^ selenious acid, 1.25 µg mL^−1^ bovine serum albumin (BSA), and 5.35 µg mL^−1^ linoleic acid), 100 ng mL^−1^ BMP‐2 (INDUCTOS), 100 ng mL^−1^ GDF5 (PeproTech), 10 ng mL^−1^ TGFβ1 (PeproTech), 1 ng mL^−1^ BMP‐6 (PeproTech) and 0.2 ng mL^−1^ FGF‐2 (R&D systems)) as previously described.^[^ ^102^ ^]^ The media were changed every second day up until day 6, where MNPs were diluted into C8 and incubated with the microtissues overnight at a final concentration of 30 µg Fe mL^−1^, prior to the magnetic biofabrication process. The effect of MNP incubation on cell proliferation was evaluated using the MTT assay kit (Abcam, ab211091) according to the manufacturer instructions. Briefly, cells were seeded in a 96‐well plate and allowed to adhere for 12 h. Afterward, they were incubated with various concentrations of MNPs for 24 h. Untreated cells served as baseline control. Following the incubation period, MTT reagent was added to each well and incubated for an additional 3 h to allow formazan crystal formation. The crystals were then dissolved using DMSO, and absorbance was measured at 570 nm using an ELISA plate reader. The cytotoxicity of MNPs on 3D cartilaginous microtissues was assessed using the LDH‐Glo Cytotoxicity Assay (Promega). Microtissues were prepared by seeding hPDCs into A400 AggreWell plates to form aggregates, followed by MNPs exposure at concentrations of 3, 30, and 60 µg Fe per mL starting on Day 1 after aggregation. Untreated microtissues served as controls. LDH release was measured at Days 1, 5, and 7. At each timepoint, culture supernatants were collected and incubated with the assay reagents as per the manufacturer's protocol. Luminescence was measured using an ELISA plate reader. Results were normalized to background luminescence from the used differentiation media and a maximum LDH release control was used to as a maximum positive measurement of cell death by lysing cells with 1% Triton‐X solution with max LDH release. Intracellular reactive oxygen species (ROS) levels were evaluated using the Dihydroethidium (DHE) Assay Kit (Abcam). hPDCs were seeded at 7000 cells per well in a 96‐well plate and allowed to adhere overnight. Cells were treated with MNPs at 3, 30, and 60 µg Fe for 24 h. Positive control wells were treated with 150 µM Antimycin A, and negative control wells were treated with 300 mM N‐acetyl cysteine for 1 h prior to the assay. DHE staining was performed as per the manufacturer's protocol, and fluorescence intensity was measured at Ex/Em = 485/570 nm using a plate reader. ROS levels were normalized to untreated controls and the background was removed and is reported as normalized fluorescence intensity. Both assays were performed in triplicate.

### Magnetic‐Guided Biofabrication

After microtissue maturation up until day 7 in the microwells and their subsequent incubation with the MNPs, a magnetic plate provided by 24 WELL BIO‐ASSEMBLER KIT (Greiner) was used to guide the magnetic driven biofabrication of assembloids. Shortly, callus microtissues were aspirated from the aggrewell and were transferred to another non‐adherent 24 well plate which was then placed on top of the magnetic plate. The magnetic field (0.4 T) in each well provided sufficient magnetic actuation to drive the bioassembly process and was maintained throughout the duration of maturation. For control samples, where MNPs were not incubated with microtissues, an agarose mold was casted by dissolving 2% agarose (Invitrogen, Belgium) in DI water and letting it solidify in 24 well plates where 3D printed rectangular inserts were used to create the mold (5 × 5 mm). Once agarose was solidified, the wells were rinsed with PBS twice and incubated at 37 °C, 5% CO_2_, and 95% humidity conditions in C8 media overnight prior use.

### In Vitro Monitoring and Viability Assay

Light microscopy was used in order to monitor both the in vitro microtissue formation as well as the assembloid post biofabrication. The samples diameter and their corresponding tissue areas were studied and measured using image analysis (ImageJ). Cell viability of assembloids was qualitatively evaluated using the LIVE/DEAD Kit (Invitrogen, USA) according to the manufacturer's instructions. In brief, assembloids were washed with PBS and subsequently incubated with 2 × 10⁻⁶ M Calcein AM and 4 × 10⁻⁶ M ethidium homodimer‐1 for 30 min at 37 °C under 5% CO₂ and 95% humidity. Fluorescent imaging was performed using an Olympus LS microscope.

### RNA Sequencing

RNA isolation from assembloid samples (N = 4, per group) was achieved by washing with PBS, prior cell lysis in 350 µL RLT lysis buffer (Qiagen, Germany) and 3.5 µL β‐mercaptoethanol (Sigma Aldrich, Germany). Samples were initially vortexed and then were stored at −80 °C. RNA sequencing and expression analysis was performed by the Genomics Core Facility in Leuven as follows: First, library preparation was performed with the Illumina TruSeq Stranded mRNA Sample Preparation Kit, according to the manufacturer protocol. RNA denaturation occurred at 65 °C followed by cooling to 4 °C. Indexed samples enabled multiplexing. The Genomics Core Leuven performed the sequencing as follows: library preparation was carried out with the Lexogen QuantSeq FWD Sample Preparation Kit, according to the manufacturer protocol. Libraries were sequenced on the Illumina HiSeq4000 sequencing system. Protocol Quality control of raw reads was implemented with FastQC v0.11.7. Adapters were filtered with Trimmomatic v0.39.^[^ ^103^ ^]^ Splice‐aware alignment was elaborated with Hisat2^[^ ^104^ ^]^ against the reference genome using the default parameters. Reads mapping to multiple loci in the reference genome were discarded. Quantification of reads per gene was performed with FeatureCounts from Subread package.^[^ ^105^ ^]^ Count‐based differential expression analysis was done with R‐based (The R Foundation for Statistical Computing, Vienna, Austria) Bioconductor package DESeq2^[^ ^106^ ^]^ following the removal of low count reads (<10), mitochondrial and ribosomal genes. Reported p‐values were adjusted for multiple testing with the Benjamini‐Hochberg procedure, which controls false discovery rate (FDR). Gene ontology analysis was performed by utilizing the EnrichR package.^[^ ^107^ ^]^ Gene lists were extracted from the gene ontology database (https://www.informatics.jax.org/vocab/gene_ontology), followed by single sample gene set enrichment analysis (ssGSEA) using Corto package.^[^ ^108^ ^]^

### In Vivo Ectopic Implantations

Subcutaneous implantation was used as a validation model to assess the assembloids capacity and autonomy to form bone tissue. Implantation took place between two distinct maturation time points (Day7 and Day21) post magnetic biofabrication (pMBF). From previous experiments, Day21 serves as the positive control of bone formation due to proper implant maturation, while Day7 assembloids were used in order to compare stimulated versus non stimulated assembloid bone forming potential at this very early time point of maturation. The produced assembloids were subcutaneously implanted in immune compromised mice (Rj:NMRInu/nu) for 4 weeks after in vivo implantation and were fixed in 4% PFA for subsequent nano‐CT and histological analysis. All animal experiments were conducted with approval from the local ethical committee for Animal Research at KU Leuven (Project license number: 128/2024). Animals were housed in compliance with the guidelines of the Animalium Leuven (KU Leuven). Last, fixed explant samples were finally used for the measurement of their mechanical properties by employing a Micro‐Tester LT (CellScale) device. Briefly, strain‐controlled compression experiments were conducted by using tungsten beams with stiffness of 4 GPa with a diameter of 0.55 mm to apply mechanical loads and record force‐displacement curves utilizing a side camera objective to record the deformation and the applied force. All experiments were carried out in pre‐warmed (37 °C) 1× PBS solution with a constant strain rate at 2% per sec and a maximum applied compressive strain of 15–30%.

### Nano‐CT

Nano‐CT (Phoenix Nanotom M, GE Measurement and Control Solutions) was used to analyze ex vivo assembloids and quantify the mineralized compartment within each explant. Scanning was performed using the following parameters: diamond target, mode 0, 500 ms exposure time, 1 frame average, no image skipping, 2400 images, and a 0.2 mm aluminum filter. Subcutaneous explants were scanned at 60 kV and 140 µA, yielding a voxel size of 2 µm. Image processing and quantification of mineralized tissue were conducted using CTAn software (Bruker micro‐CT, BE) through automatic segmentation, 3D space filling, and spot despeckle algorithms. The percentage of mineralized tissue was determined relative to the total explant volume. For 3D visualization, CTvox (Bruker micro‐CT, BE) was utilized.

### Histology and Immunostaining

Retrieved subcutaneous explants were fixed overnight in 4% PFA, followed by decalcification in ethylenediaminetetraacetic acid (EDTA)/PBS (pH 7.5) at 4 °C for 10 days. Samples were then embedded in paraffin and sectioned into 5 µm thick slices. Histological assessment was performed using Alcian Blue, Masson's Trichrome, and Safranin O staining. Immunohistochemistry was conducted on PFA‐fixed assembloid sections to detect Osterix, SHG, and COL2. Antigen retrieval was carried out by incubating the sections in 0.2 M sodium citrate solution at 70 °C for 30 min. Endogenous peroxidase activity was quenched with 3% H₂O₂ for 10 min. Sections were then blocked for 1 h in a solution containing 3% BSA in FBS and incubated overnight at 4 °C with primary antibodies: human Osterix (R&D Systems, MAB7547; 1:300 dilution) and rabbit anti‐collagen type II (Merck Millipore, AB761; 1:50 dilution). After primary incubation, slides were blocked again and treated with Alexa 488 anti‐mouse secondary antibody (Thermo Fisher Scientific, A11001; 1:500 dilution). Imaging of stained histological sections was performed using a ZEISS LSM 510 META confocal microscope at the KU Leuven Cell Imaging Core Facility. Additionally, in vitro and ex vivo samples, as well as liver and spleen sections, were stained with Prussian Blue to visualize iron deposition within the tissues

### Hysteresis Measurement of Magnetic Assembloids

Magnetization measurements were performed using a SQUID magnetometer (Quantum Design MPMS3) at room temperature (300K). Bone tissue explants were divided into two groups: control tissues without MNPs treatment and experimental tissues treated with MNPs. Each group contained two types of dried samples: in vitro and *ex vivo* explants in different corresponding timepoints (Day7 and Day21). The control tissues served as a baseline for the magnetization signal originating from the intrinsic properties of the bone tissues. Measurements were carried out by applying a magnetic field ranging from −7 Tesla (T) to +7 T, and the resulting magnetic moment was recorded as a function of the applied magnetic field. The saturation magnetic moment was determined for each sample at 7 T. The experimental tissues were measured and compared to their corresponding control tissues to remove the background magnetic contribution. The corrected magnetization was calculated by subtracting the magnetization of the control tissues from that of the experimental tissues. Additionally, a pure dried MNP powder sample of known weight was measured to determine the intrinsic magnetic properties of the nanoparticles in isolation. A palladium (Pd) rod was also measured as a non‐magnetic reference to validate the system's sensitivity and ensure that background signals were negligible. Hysteresis curves, representing the magnetic moment (emu g^−1^) versus the applied magnetic field (T), were plotted for all samples. The baseline‐corrected magnetization curves were used to assess the contribution of the MNPs to the overall magnetic behavior of the experimental tissues.

### Computational Model of Magnetic Bioassembly

In this study, we employed Mpacts, a particle‐based simulation software, to model the dynamics of magnetic cartilaginous microtissues. The simulations were visualized using Paraview software. Each microtissue was represented as a spherical particle. Initially, the particles were randomly positioned within a cylindrical well to mimic the experimental setup. The number and radius of the microtissues in the simulations were determined based on experimental measurements. These measurements were obtained by photographing a well containing microtissues and utilizing an object detection algorithm trained with ilastik software to automatically identify and measure the radius of the microtissues, as well as to count the number of microtissues in the well. This was essential for accurate simulation of the magnetic biofabrication process within one well of microtissues. The equations describing our system are explained in detail below:

The dynamics of a microtissue *** i*** with position ***x***_***i***_, velocity ***v***_**i**_, and radius ***r***_***i***_ are governed by the overdamped force balance: $F_{d,i}+\sum_{\delta_{\mathrm{ij}}\geq 0}F_{c,\mathrm{ij}}+F_{m,i}+F_{g,i}+\sum_{\delta_{\mathrm{ij}}\geq 0}\;F_{k,\mathrm{ij}}=0$. Here, ***F***_***c***, ***ij***_ is the drag force due to contact between microtissue ***i*** and microtissue ***j*** and between a microtissue and the well, which is dependent on their overlap ***δ***_***ij***_. ***F***_***g***, ***i***_ is the gravitational force and ***F***_***d***, ***i***_ is the Stokes drag force acting on spheroid ***i***. ***F***_***k***, ***ij***_ is the potential force between two colliding microtissues *** i*** and *** j***, modeled as a Hertzian repulsion force. ***F***_***m***, ***i***_ is the magnetic force on the microtissue ***i***, calculated by scaling the number of MNPs absorbed on the periphery of a spheroid with the magnetic force calculated for a single nanoparticle, based on the methodology outlined in.^[^ ^109^ ^]^ The equation of motion for all particles is a system of linear equations **C** **v**  =** **F**, which is solved for microtissue velocities at each time. To simulate dynamics, a semi‐implicit numerical integration scheme was employed using overdamped equations of motion. The model was validated against experimental observations by measuring the aggregation area over time. Experimentally, this measurement was performed using ImageJ, while the simulation data was processed using Python. Additionally, the rate profile at a distance from the magnet was evaluated by normalizing the rate of microtissue movement at each position with their radius and taking the average over microtissues. In experiments, this was achieved using Tracker software, while in simulations, it was measured using Python. The mechanical stress within the aggregate was further assessed by calculating the radial stress in silico at different positions of the microtissues from the center of the magnet. Each tissue spheroid was represented as a single sphere. Initially, the particles were randomly positioned within a cylindrical well to mimic the experimental setup. The number and radii of the spheroids in the simulations were determined based on experimental measurements. On these spheres, various body forces and contact forces act, which are described in the following sections.

### Magnetic Force

The magnetic forces acting on single spheroids are calculated by scaling the magnetic force acting on a single MNP with the number of MNPs absorbed on the surface of the spheroid. To begin, the magnetic force acting on a single MNP is calculated using

(1) $$F_{m,NP}=\mu_{0}\;V_{\mathrm{NP}}f\left(\left|H\right|\right)\left(H\cdot \nabla \right)H$$

defined by Wirthl et al. (2023). This formula enables the calculation of the magnetic force acting on a single nanoparticle. The formula for the forces acting on a spheroid coated with multiple MNPs is

(2) $$F_{m,i}=N_{\mathrm{NP},i}\;\mu_{0}V_{\mathrm{NP}}f\left(\left|H\right|\right)\left(H\cdot \nabla \right)H$$

The volume (*V*_NP_) of the nanoparticle is determined by $V_{\mathrm{NP}}=\frac{4}{3}\;\pi r_{NP}^{3}$, leveraging the known radius *r_NP_* of the nanoparticles. *N*_*NP*,*i*_ is the number of nanoparticles in spheroid *i* and the magnetization model

(3) $$f\;\left(\left|H\right|\right)=\left\{\begin{array}{c} 3\;\left|H\right|<\frac{1}{3}M_{\mathrm{NP}}\\ \frac{M_{\mathrm{NP}}}{\left|H\right|}\;\left|H\right|\geq \frac{1}{3}M_{\mathrm{NP}} \end{array}\right.$$

can be employed, given the knowledge of the particles' saturation magnetization which is equal to 60 emu g^−1^. Finally, ***H*** denotes the magnetic field strength. The precise number of nanoparticles in each spheroid is unknown. However, the total mass of nanoparticles added *m*_NPtot_ to all spheroids is known. Using this mass, the total number of nanoparticles

(4) $$N_{\mathrm{NPtot}}=\frac{m_{\mathrm{NPtot}}}{V_{\mathrm{NP}}\rho_{\mathrm{NP}}}$$

can be calculated. The number of nanoparticles on the surface of the spheroid can be found by scaling the number of nanoparticles with the area of the individual spheroid, where *N_NPtot_* is the total number of nanoparticles and ρ_*NP*_ is the density of the nanoparticles. This is an assumption that is made since the spheroids are coated with MNPs on their periphery. It is assumed that not all the nanoparticles are absorbed by the spheroids, and the remaining nanoparticles remain in the media and do not contribute to the calculation of the magnetic force exerted on the spheroids. Therefore, an absorption factor α is defined. Thus, one can calculate the number of nanoparticles in a spheroid by

(5) $$N_{\mathrm{NP}}=\alpha \frac{N_{\mathrm{NPtot}}A_{i}}{A_{\mathrm{tot}}}$$

where *A_i_* represents the surface area of spheroid *i*, while *A_tot_* denotes the total surface area of the spheroids in the well. However, due to the inherent randomness in biological systems, precise predictions of behavior using exact numbers can be challenging. Hence, the final count of nanoparticles within the spheroid was sampled from a normal distribution. This distribution had a mean equivalent to the number of nanoparticles expected based on the previous equation, and a standard deviation equal to half of the mean, reflecting the inherent variability in the system.

### Drag Force

Due to the spheroid's motion through the media, it encounters a drag force, as per Stokes' law

(6) $$F_{d,i}=-6\pi \eta_{m}r_{i}v_{i}$$

where η_*m*_ represents the viscosity of the medium. The direction of this force is opposite to the movement of the spheroid in the medium.

### Gravitational Force

The gravitational force acting upon the spheroids, owing to their mass already subtracted by the upward buoyancy force, is determined by:

(7) $$F_{g,i}=\frac{4}{3}\;\pi r_{i}^{3}\left(\rho_{s}-\rho_{m}\right)g$$

where ρ_*s*_ is the density of the spheroids (1014 kg m^−^^3^), ρ_*m*_ is the density of the medium, assumed to be equal water, namely 1000 kg m^−^^3^. ***g*** is gravitational acceleration. In the x‐ and y‐directions, ***g*** is zero, while it takes a value of 9.81 m s^−^^2^ in the negative z‐direction.

### Contact Models

Collision forces occur between the spheroids themselves and between the well and the spheroid. In the model, the well is represented as a 3D object, allowing the spheroids to move freely within it. This is achieved by creating an STL file using Autodesk Fusion 360. The resulting 3D object is then represented as a mesh of triangles. Consequently, the contact forces with the well are modeled between the spheroids and the triangle within the model.

For the collision of spheroid *i* with spheroid *j*,  their overlap distance is:

(8) $$\delta_{ij}=r_{i}+r_{j}-\left|\left|x_{i}-x_{j}\right|\right|$$

and their normal unit vector of the contact is

(9) $$\hat{n_{\mathrm{ij}}}=\frac{x_{i}-x_{j}}{\left|\left|x_{i}-x_{j}\right|\right|}\;$$

For the contact between the cylindrical well with outward normal $\hat{n_{w}}$ and spheroid *i*, the overlap distance is

(10) $$\delta_{iw}=r_{i}\;-\left(x_{i}-x_{w}\right)\cdot \hat{n_{w}}$$

with *x_w_* the position at any point in the plane. Thus, the contact normal unit vector is equal to

(11) $$\hat{n_{iw}}=\hat{n_{w}}\;$$

### Contact Forces

The aggregation of spheroids inside a magnetic field occurs on a timescale of seconds. Due to the large magnetic forces and the gravitational forces, very fast dynamics are achieved. Therefore, we can neglect active cell mechanical processes. Additionally, stochastic (thermal) forces can be neglected for these spheroids since they have a radius larger than 100 µm. Contact forces are computed between two colliding pairs *ij* that have overlap distance of δ_*ij*_ ≥ 0. There are two types of contact forces, potential forces and drag or friction forces. Potential forces depend on the overlap between two particles and are equal to ***F***_*k*,*ij*_(δ_*ij*_). The collision of spheroids is modelled as a repulsive interaction between two elastic bodies. The potential interaction force which is described by the Hertz model is:

(12) $$F_{H,ij}=\frac{4}{3}\;\hat{E_{0}}\sqrt{\hat{r_{ij}}}\delta_{ij}^{3/2}\hat{n_{ij}}$$

with effective contact radius

(13) $$\hat{r_{ij}}=\frac{r_{i}r_{j}}{r_{i}+r_{j}}$$

and instantaneous effective contact stiffness $\hat{E_{0}}.$ The contact area is *S_ij_* and is based on the Hertz contact radius:

(14) $$S_{ij}=\pi \delta_{ij}\hat{r_{ij}}$$

Moreover, the coefficients of normal and tangential friction, *c_n_* and *c_t_*, respectively, represent the effective friction resulting from wet and rolling friction, assuming that rotational particle motion is ignored, as well as normal viscous contact dissipation.

Next, the drag forces, in this case between the well and the spheroid

(15) $$F_{c,ij}=-S_{ij}\left[c_{t}v_{ij}+\left(c_{n}-c_{t}\right)\left(v_{ij}\cdot {\hat{n}}_{ij}\right){\hat{n}}_{ij}\right]$$

which are dependent of the relative velocity ***v**_ij_*, with ***v**_ij_* = ***v**_i_*  − ***v**_j_* of the two particles in contact. Here, *c_n_* and *c_t_* are the normal and tangential wet friction constants, and *S_ij_* is the contact area between particles *i* and *j* The contact force for both particles is of equal magnitude with an opposite sign for both contacting particles in a contact pair *ij*, thus ***F**_ij_* =   − ***F**_ji_*.

### Equations of Motion

Combining all these forces together into one equation of motion for particle *i* which is given by:

(16) $$-F_{d,i}-\;\sum_{\delta_{ij}\geq 0}F_{c,ij}=F_{m,i}+F_{g,i}+\sum_{\delta_{ij}\geq 0}F_{k,ij}$$

The left‐hand side of the equation comprises all velocity dependent forces, while the right‐hand side contains the body and contact forces. The equation of motion for all particles can then be written as a system of linear equations

(17) Cv=F

since all terms depending on velocity are linear. Here ***C*** is a 3 *n*  × 3 *n* matrix where the diagonal elements are formed by the medium drag and the other elements are formed by the contact pairs *i* and *j* and $v={\left[v_{1},\text{…},v_{i},\text{…},v_{n}\right]}^{T}$ and $F={\left[F_{1},\text{…},F_{i},\text{…},F_{n}\right]}^{T}$ are the state vectors.

Thus, for example, a diagonal element is defined as:

(18) $$c_{ii}=6\pi \eta_{m}\;r_{i}\;I+\sum_{\delta_{ij}\geq 0}c_{n}\hat{n_{ij}}\hat{n_{ij}^{T}}+c_{t}\left(I-\hat{n_{ij}}\hat{n_{ij}^{T}}\right)$$

and an off‐diagonal element as:

(19) $$c_{ij}=c_{ji}=-\sum_{\delta_{ij}\geq 0}c_{n}\hat{n_{ij}}\hat{n_{ij}^{T}}+c_{t}\left(I-\hat{n_{ij}}\hat{n_{ij}^{T}}\right)$$

***C*** is a sparse, positive definite and a symmetric matrix, therefore this system can be solved by the conjugate gradient method.

### Time Integration

The linear system with the equations of motion is solved by using semi‐implicit time integration. The particle velocities get updated by:

(20) $$C\left(c,k\lambda \delta t\right)v_{t+\delta t}=F_{t}$$

here ***c*** and ***k*** are the gradient ***c**_ij_* = ∇_*v*_ ***F**_ij_* and ***k**_ij_* = ∇_*x*_ ***F**_ij_*, and λ is the degree of implicitness in the semi‐implicit scheme. For example, for potential forces, we can extract ***k**_ij_* as

(21) $$k_{ij}=\frac{dF_{k,ij}}{d\delta_{ij}}\;\hat{n_{ij}^{T}}$$

After updating these velocities, next the positions are also updated as follows:

(22) $$x_{t+\delta t}=x_{i,t}\;+\delta t\;v_{i.t+\delta t}+\sqrt{2D}\;dW_{t}$$

In this equation, thermal diffusion is introduced with *D* the diffusivity. Where *d**W**_t_* is the increment of a standardized Wiener process in time *t*.

### Statistical Analysis

All experiments were performed with at least four replicates per condition unless otherwise stated. Data are presented as mean ± standard deviation (SD) or mean ± standard error of the mean (SEM), as specified in the figure legends. Prior to statistical analysis, data were assessed for normality using the Shapiro‐Wilk test and for homogeneity of variances using Levene's test. If data did not meet normality or homoscedasticity assumptions, appropriate transformations were applied. Normalization of data followed the manufacturer's guidelines for each assay. For comparisons between two groups, a two‐tailed unpaired Student's *t*‐test was used. For multiple group comparisons, one‐way or two‐way analysis of variance (ANOVA) was performed, followed by Bonferroni's or Tukey's post‐hoc test to account for multiple comparisons. Statistical significance was determined with an alpha value of 0.05, and *p*‐values are reported as *p < 0.05, **p < 0.01, ***p < 0.001, and ****p < 0.0001. Sample sizes (N) for each experimental group and the statistical test applied are specified in the figure legends. All statistical analyses and graphical representations were conducted using GraphPad Prism (version 10, GraphPad Software, San Diego, CA, USA).

## Conflict of Interest

The authors declare no conflict of interest.

## Supporting information



Supporting Information

Supplemental Video 1

Supplemental Video 2

Supplemental Video 3

## Data Availability

The data that support the findings of this study are available in the supplementary material of this article.
