# Supplementary material for: 4D Biofabrication of Magnetically Augmented Callus Assembloid Implants Enables Rapid Endochondral Ossification via Activation of Mechanosensitive Pathways
Source: Adv Sci (Weinh). 2025 Feb 25;12(15):2413680. doi: 10.1002/advs.202413680 (PMC12005758; doi:10.1002/advs.202413680)
Supplement: Supplementary file 1 — Supporting Information [file ADVS-12-2413680-s001.docx]

**Supporting Information**

**Supplementary Figure S1**

*
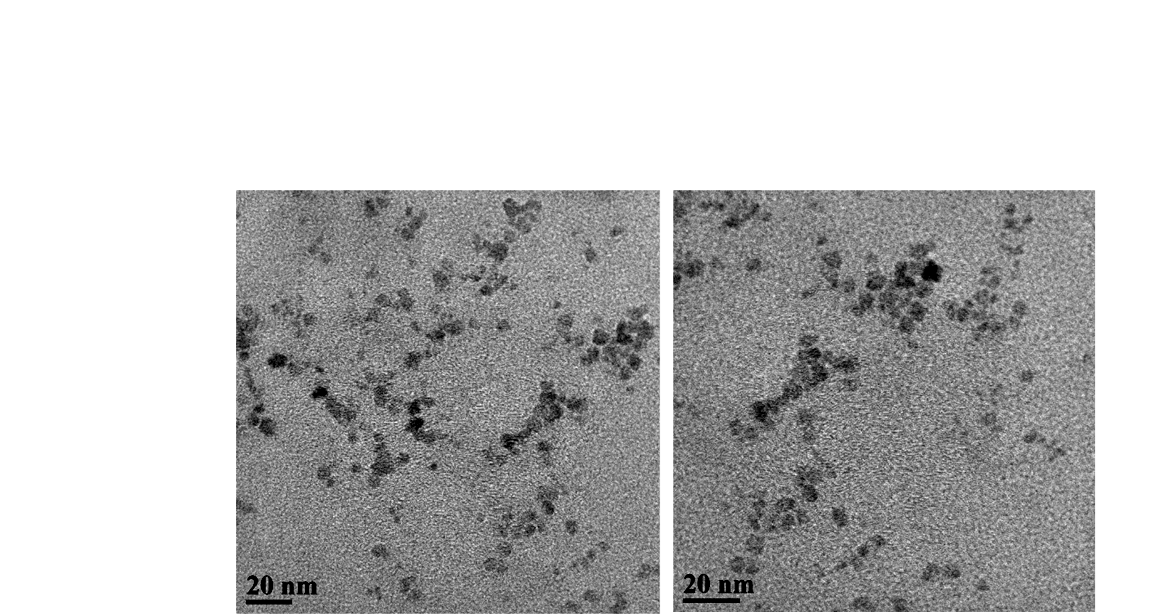
*

**Figure S1. TEM images of MNPs.** TEM images of IONPs-CA with relatively low particle concentrations

**
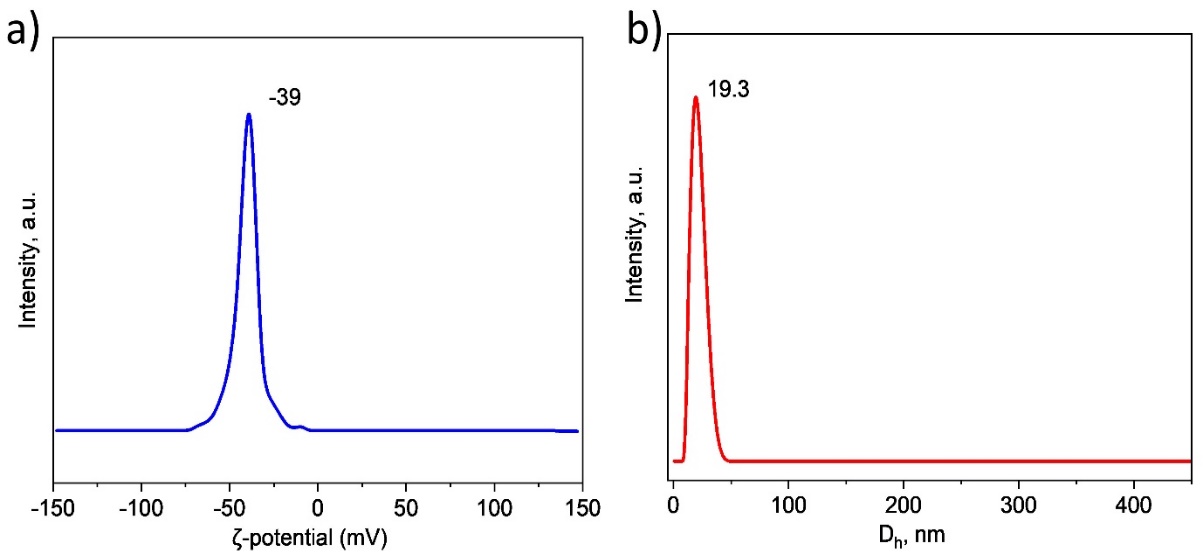
Supplementary Figure S2**

**Figure S2. MNPs characterization.** a) ζ-potential of IONPs-CA and b) DLS graph of IONPs-CA

**
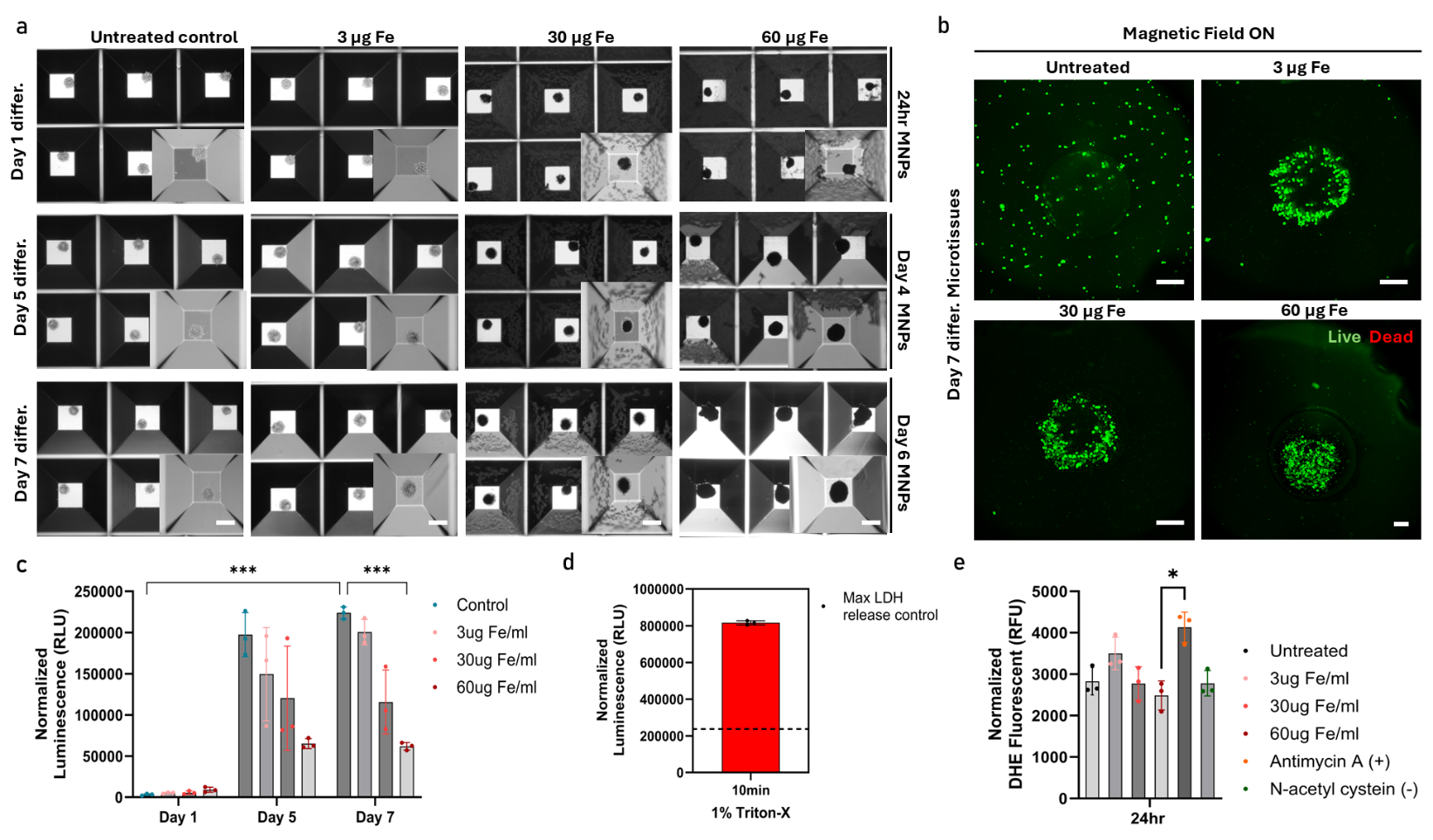
Supplementary Figure S3**

**Figure S3. Long-term LDH assay on 3D cartilaginous microtissues.** **a)** Microscope images of untreated microtissues and microtissues with varying MNP loads (3, 30, and 60 μg Fe) captured on Days 2, 5, and 7.**b)** Assessment of cell viability after 7 days using MBF with Live/Dead staining, showing no significant hindrance to viability across conditions. c**)** LDH release assay results indicating decreased LDH release with higher Fe loading concentrations over time. **d)** Positive control showing the maximum LDH release in the 3D system, with RLU values used as a reference. The dotted line represents the highest LDH levels observed in untreated samples on Day 7. **e)** ROS assay results revealing reduced DHE RFU values corresponding to increased Fe loading. **Statistical Analysis:** For panel (c), Statistical analysis was performed using two-way ANOVA with Bonferroni correction (N = 3), with a significance threshold of *** **p < 0.001**. For panel (e), a t-test was conducted to identify significant differences, with a significance level of * **p < 0.05**. Scalebar: a) 500μm, b)1mm.

**Supplementary Figure S4**


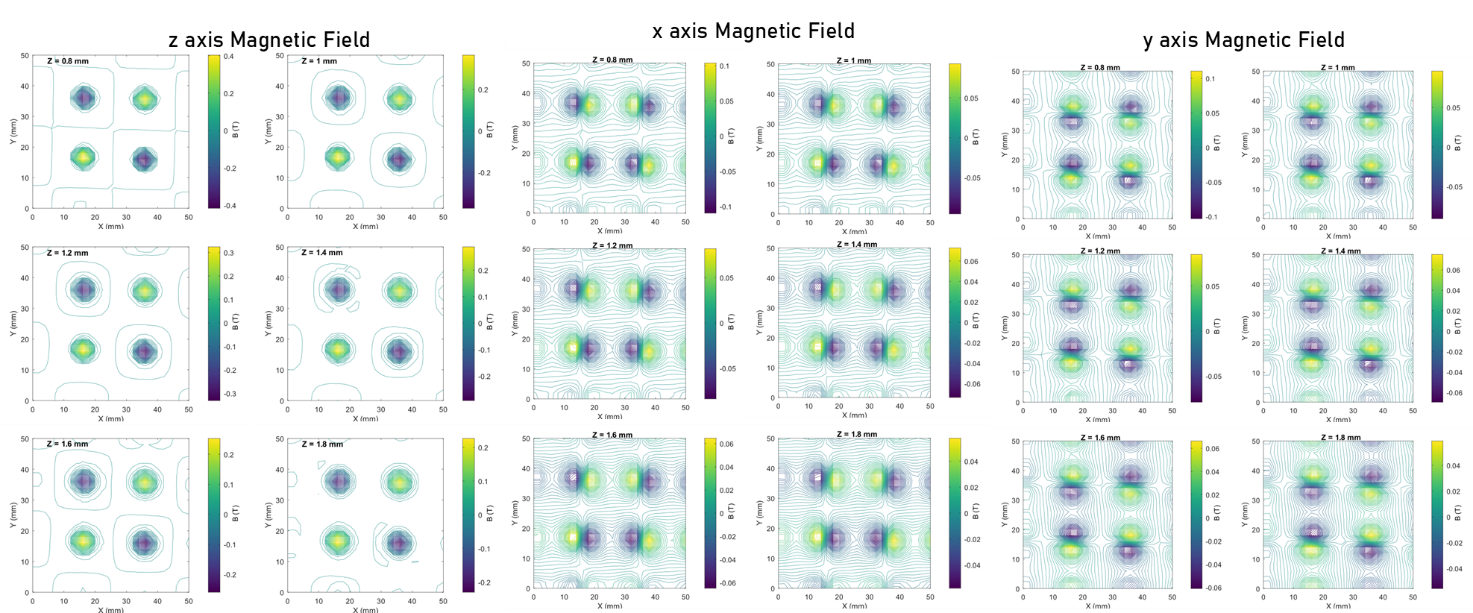


**Figure. S4. 3D magnetic field mapping across z,x and y axis.** Resolution is 0,1mm and the increasing z-step was 0.2mm.

**Supplementary Figure S5**

**
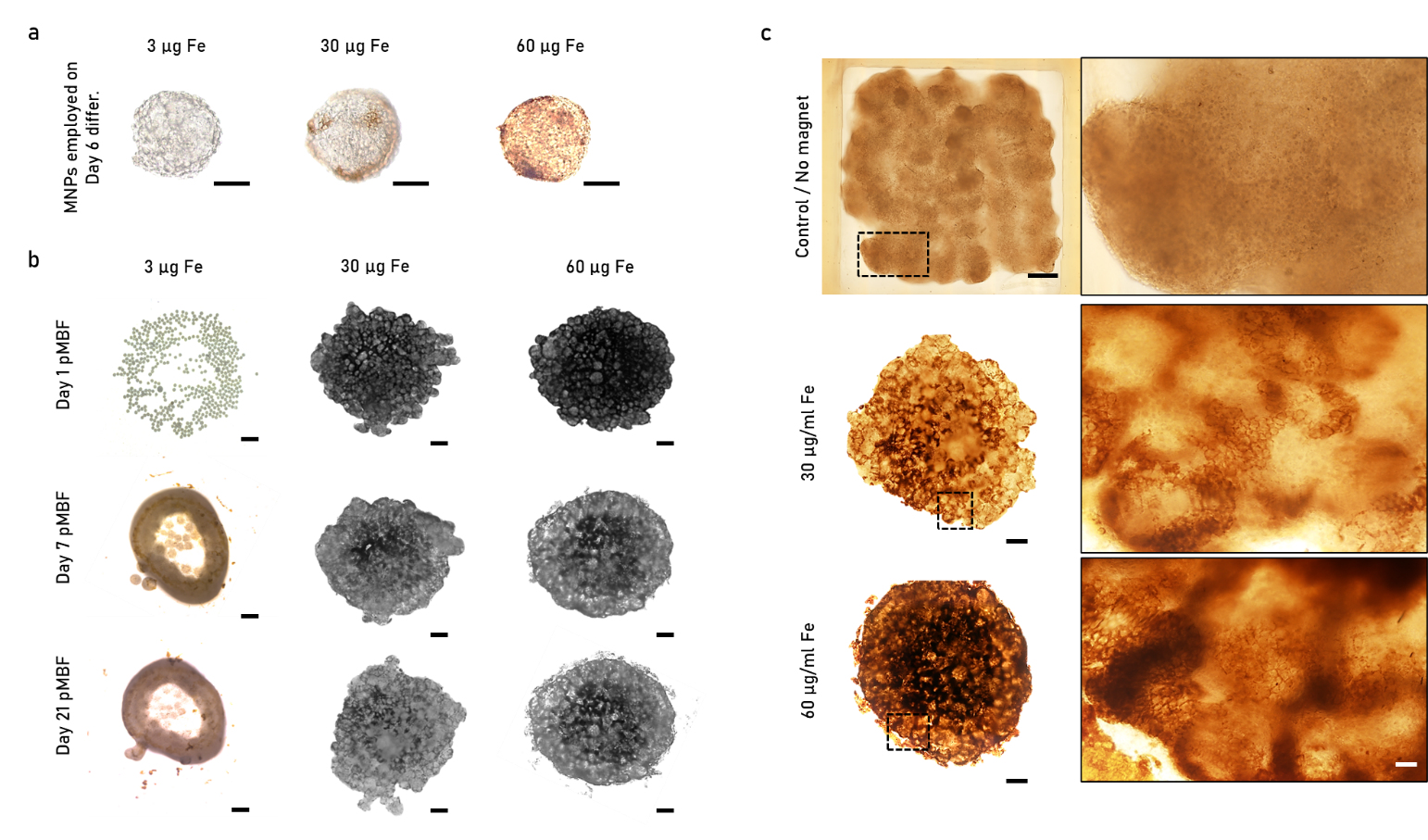
**

**Figure. S5. Magnetic driven assembly and fusion of microtissues with different MNPs loading.** a) Individual microtissues on Day 7 after 24hr incubation with MNPs with different Fe loadings (3, 30 and 60μg Fe). b) Images showing overtime the culture and growth of the differentially Fe loaded MCAs and c) Microscope images on Day 7 between control, 30 and 60 μg of Fe MACs. Scalebars: a) 500μm, b) 500μm and 50μm.

.

**
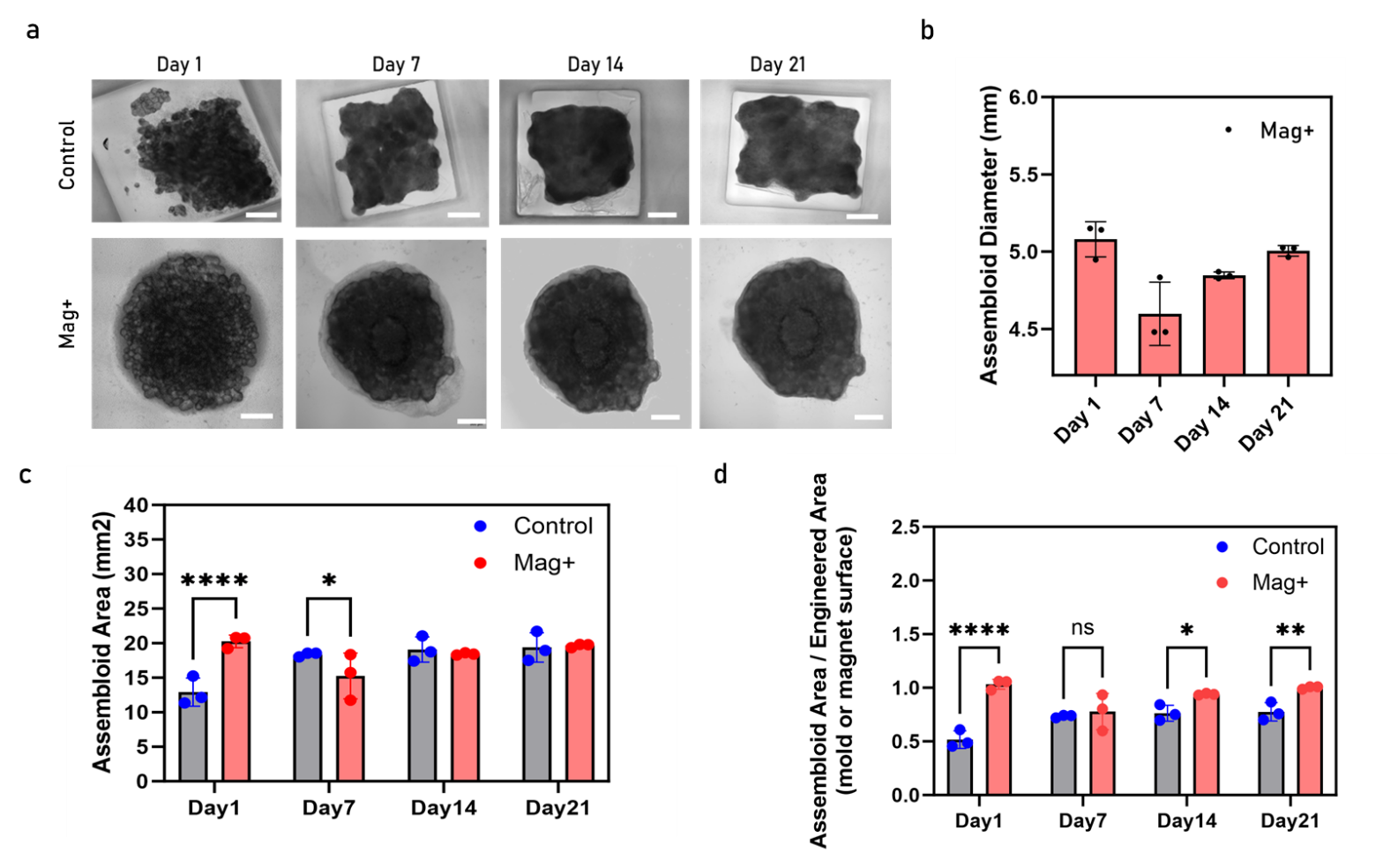
Supplementary Figure S6**

**Figure. S6. In vitro maturation and growth of assembloids.** a) Overtime monitoring of the assembloids with microscope. b) Measurement of the MCAs sample diameter measured in 3 replicates, showcasing 5mm diameter assembly on Day1, which is decreased due to fusion kinetics on Day 7 and starts to grow back to 5mm in diameter assembloid again on Day21. c) Assembloid Area (mm^2^)plot highlighting the main differences between the two groups. d) Measurements showing the area of assembloids divided by the area of the mold or magnet used in their assembly, highlighting the significant impact of magnetic guidance towards biofabrication area. Statistical analysis was performed using two-way ANOVA with Bonferroni correction (N = 3), with a significance threshold of * **p < 0.05**; ****p<0.01; ***** **p < 0.001**.


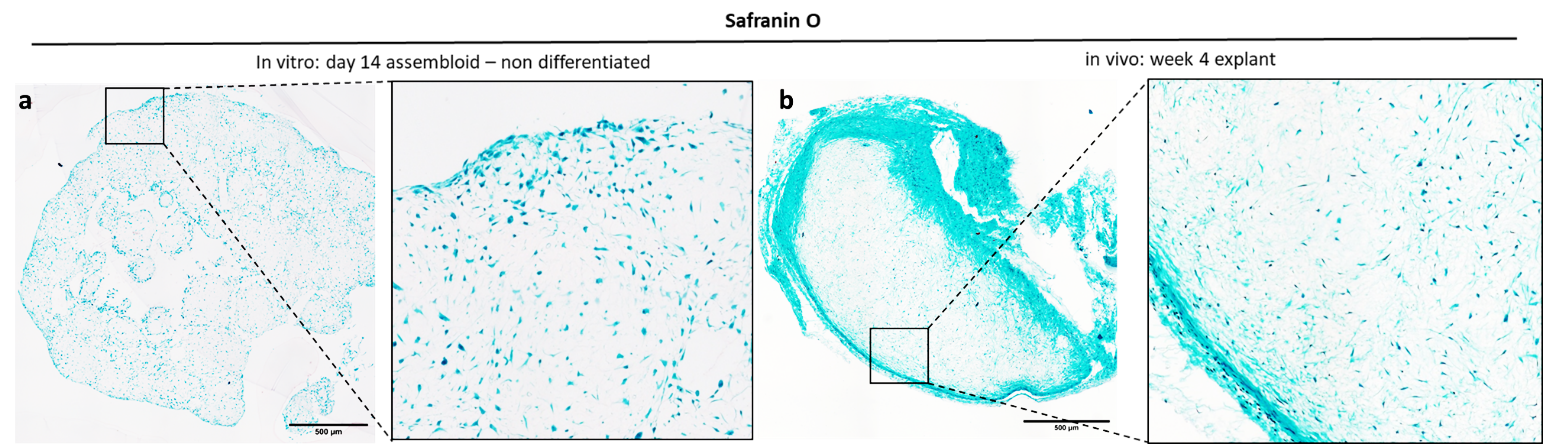
**Supplementary Figure S7**

***Figure S7: Negative control of bone formation through endochondral ossification.*** *a) Safranin O-stained histological image showing an undifferentiated, fibrotic implant on day 14, indicating the absence of successful differentiation through negative Safranin O-stained areas (lack of GAGs and cartilaginous ECM). b) Corresponding histological section of an explant retrieved after 4 weeks ectopically in vivo, demonstrating a failed bone remodeling process due to absence of cartilaginous template, characterized by disrupted tissue organization counterstained with Fast Green. Scalebar: 500μm.*

**Supplementary Figure S8**

**
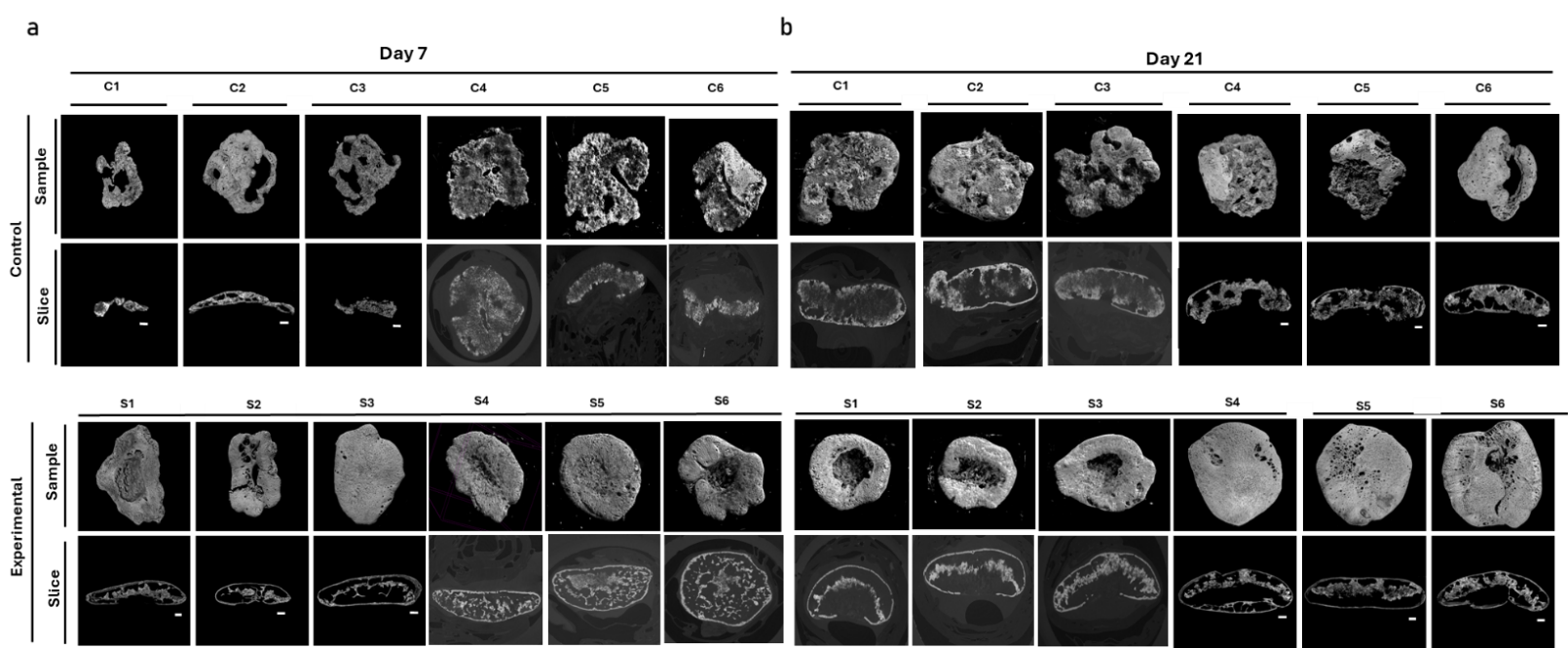
**

**Figure.S8. μCT 3D reconstructed images of the 6 replicate explants per group used in this study and their corresponding implantation timepoint.** All explants were kept 4 weeks in vivo. Scalebars: 250μm.

**
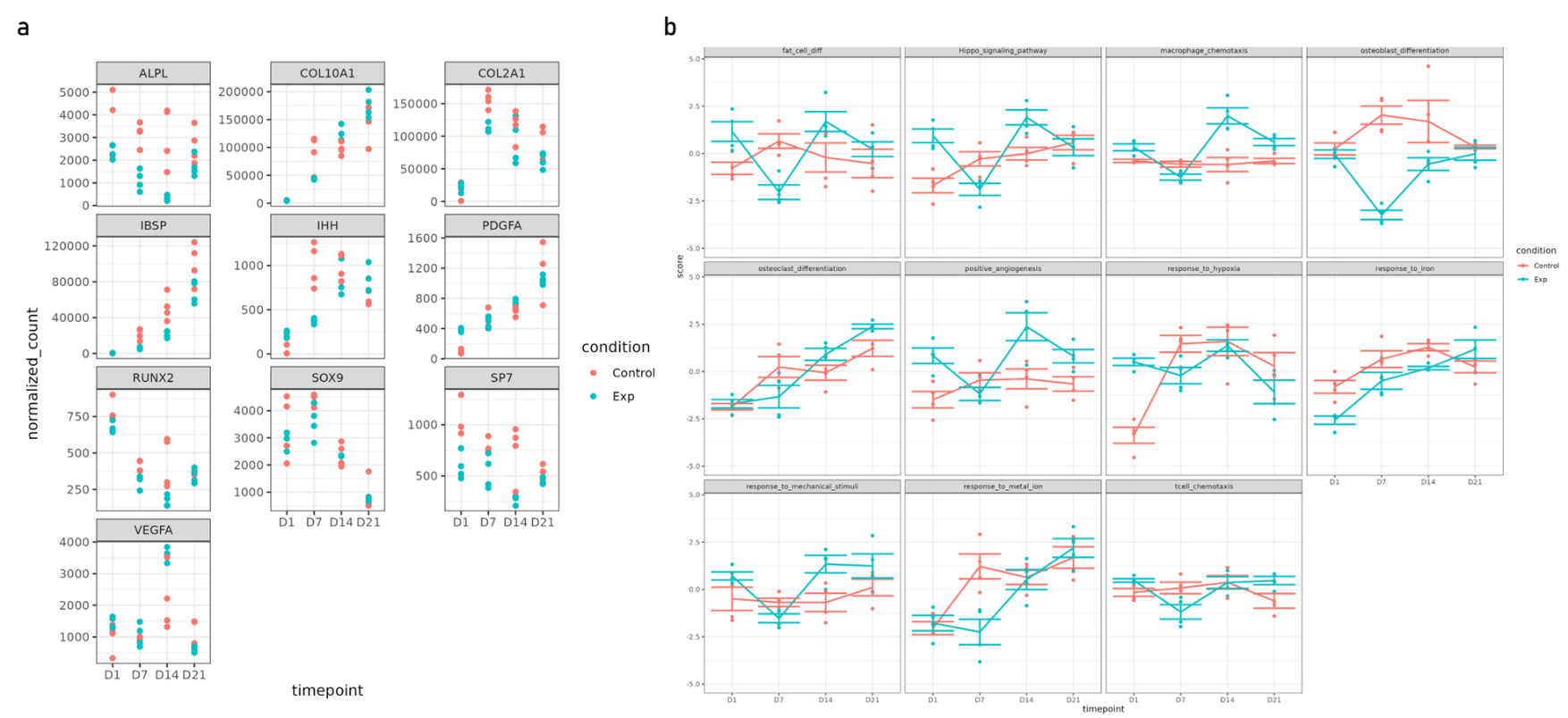
Supplementary Figure S9**

**Figure. S9. RNA sequencing analysis.** a) Hypertrophy gene panel showcasing minimal expression differences in hypertrophic commitment. b) Extended panel of ssGSEA plots overtime.
