# Supplementary figures and images for: 4D Biofabrication of Magnetically Augmented Callus Assembloid Implants Enables Rapid Endochondral Ossification via Activation of Mechanosensitive Pathways
Source: Adv Sci (Weinh). 2025 Feb 25;12(15):2413680. doi: 10.1002/advs.202413680 (PMC12005758; doi:10.1002/advs.202413680)

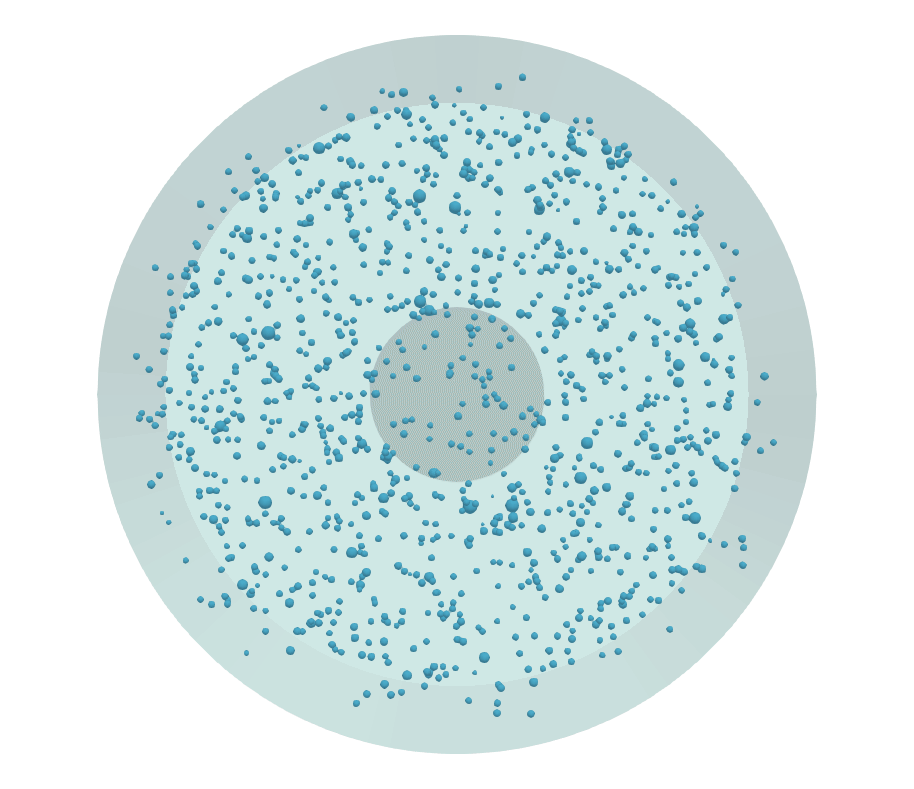

Supplement: Supplementary file 3 — Supplemental Video 2 [file ADVS-12-2413680-s004.gif]
